# Multi-platform Approach for Microbial Biomarker Identification Using *Borrelia burgdorferi* as a Model

**DOI:** 10.3389/fcimb.2019.00179

**Published:** 2019-06-11

**Authors:** Kathryn J. Pflughoeft, Michael Mash, Nicole R. Hasenkampf, Mary B. Jacobs, Amanda C. Tardo, D. Mitchell Magee, Lusheng Song, Joshua LaBaer, Mario T. Philipp, Monica E. Embers, David P. AuCoin

**Affiliations:** ^1^DxDiscovery, Inc., Reno, NV, United States; ^2^Department of Microbiology and Immunology, Reno School of Medicine, University of Nevada, Reno, NV, United States; ^3^Division of Bacteriology and Parasitology, Tulane National Primate Research Center, Tulane University Health Sciences Center, Covington, LA, United States; ^4^Center for Personalized Diagnostics, Biodesign Institute, Arizona State University, Tempe, AZ, United States

**Keywords:** microbial biomarker discovery, Lyme disease, early diagnostic, antibody response, *Borrelia burgdorferi*

## Abstract

The identification of microbial biomarkers is critical for the diagnosis of a disease early during infection. However, the identification of reliable biomarkers is often hampered by a low concentration of microbes or biomarkers within host fluids or tissues. We have outlined a multi-platform strategy to assess microbial biomarkers that can be consistently detected in host samples, using *Borrelia burgdorferi*, the causative agent of Lyme disease, as an example. Key aspects of the strategy include the selection of a macaque model of human disease, *in vivo* Microbial Antigen Discovery (InMAD), and proteomic methods that include microbial biomarker enrichment within samples to identify secreted proteins circulating during infection. Using the described strategy, we have identified 6 biomarkers from multiple samples. In addition, the temporal antibody response to select bacterial antigens was mapped. By integrating biomarkers identified from early infection with temporal patterns of expression, the described platform allows for the data driven selection of diagnostic targets.

## Introduction

Successful treatment of many infectious diseases relies on the detection of a pathogen or secreted microbial biomarker early during infection. Early effective treatment is critical to limit damage caused directly by the pathogen or due to the host immune response (Goletti et al., 2018; Wiersinga et al., 2018). A necessary component for diagnosis is selection of an appropriate microbe-specific marker that is indicative of disease (microbial biomarker), or combinations of biomarkers that are present at detectable levels at distinct stages of disease. However, microbes or microbial diagnostic biomarkers contained in patient samples are often at low concentrations during acute infection. While selection of such microbial biomarkers may be done *in silico* for well-characterized bacteria and less genomically complex microbes, like viruses, the prediction of diagnostic biomarkers for bacteria possessing complex genomes and those that undergo antigenic variation are likely to require well-implemented wet lab approaches. Approaches that consider the composition of antigens expressed *in vitro* often differs from those expressed *in vivo*.

The clearance of organisms from blood and other accessible biological fluids along with the variable intensity of the immune response to *Borrelia burgdorferi* biomarkers make the diagnosis and treatment of Lyme disease an ongoing challenge (Embers et al., 2016; Schutzer et al., 2018). The difficulties associated with detection of *Borrelia burgdorferi* made the pathogen an ideal case for developing a multi-platform approach for the detection of a low abundance pathogen from host samples. Analyses of the number of Lyme disease serodiagnostic tests performed at clinical testing centers, and the subsequent results, allowed for an estimate of >300,000 cases of Lyme disease in the U.S. each year (Hinckley et al., 2014). The current method for diagnosis recommended by the CDC is a two-tier serologic assay consisting of an enzyme-linked immunosorbent assay (ELISA) followed by an immunoblot (Moore et al., 2016; Branda et al., 2017). Administration of the second tier of the test (IgG immunoblot), is not recommended until several weeks post-infection due to its reliance on a detectible IgG antibody response. An IgM immunoblot can be used earlier in disease, with the understanding that the result should not be used solely for diagnosis (Centers for Disease Control and Prevention, 2015; Branda et al., 2017; U.S. Department of Health and Human Services, 2017). Without treatment early during infection, the bacteria may disseminate, leading to the characteristic rheumatologic, cardiac and neurological manifestations of Lyme disease. The clinical features of Lyme disease can be broken down into distinct stages. Early disease is characterized by the tell-tale Erythema Migrans (EM) rash; however, an EM only presents in 60–80% of patients (Steere, 1989). Early disseminated and late infection phases can be characterized by persistent neurological signs and/or arthritis (Steere, 1989, 2001; Steere et al., 2016). Early diagnosis of Lyme disease, leading to the early initiation of treatment, can limit its progression into the late stages of disease and therefore, reduce human morbidity.

**Figure 1:**
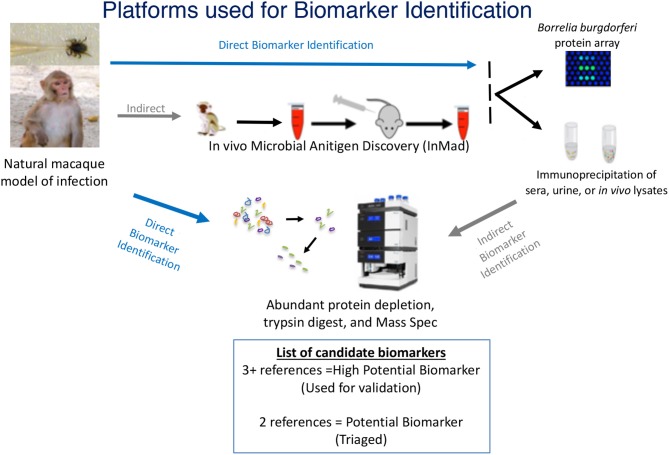
Multiplatform Approach for Microbial Biomarker Identification—Microbial biomarkers were directly or indirectly identified from samples collected from an infected host, in the case of this study, a macaque model of infection. Techniques used for direct detection of microbial biomarkers included mass spectrometry (MS) of concentrated or enriched samples and protein array. Indirect detection included the InMAD strategy coupled with protein array and immunoprecipitation-coupled MS. Identified biomarkers were categorized based upon the number of times each was identified by either direct or indirect analysis. The image of the mass spectometer was copied from the thermo website (https://www.thermofisher.com/order/catalog/product/ULTIM3000RSLCNANO).

The goal of this study was to develop a standardized approach for identification of microbial antigens that can be detected early during disease and that can be applied to most, if not all infectious diseases. To meet this goal, a discovery-based strategy was designed to identify antigens specific to *B. burgdorferi* in sera or urine of infected animals. A proteomic approach was selected for the identification of proteins that could be found in samples, proteins were detected either through direct analysis via mass spectrometry (MS) or through indirect analysis, which included an enrichment step using immunoprecipitation prior to MS. Proteomic approaches were used in conjunction with the *in vivo* Microbial Antigen Discovery (InMAD) platform, in which healthy mice are immunized with filtered serum collected from an infected host (Figure 1) (Nuti et al., 2011). The InMAD approach was included in the study as it allows for the generation of antibodies in a secondary host to the array of circulating microbial proteins or polysaccharides present at a specific point in an infection of the primary host. Finally, protein arrays were used to validate that the host, either mouse or macaque, had been exposed to an antigen, as well as to begin to map the temporal pattern of biomarker display.

## Materials and Methods

### Animal Welfare Statement

Practices in the housing and care of non-human primates and rats conformed to the regulations and standards of the Public Health Service Policy on Humane Care and Use of Laboratory Animals, and the Guide for the Care and Use of Laboratory Animals. The Tulane National Primate Research Center (TNPRC) is fully accredited by the Association for the Assessment and Accreditation of Laboratory Animal Care-International. The Tulane University Institutional Animal Care and Use Committee approved all animal-related protocols, including the infection and sample collection from NHPs. All animal procedures were overseen by veterinarians and their staff. Rat surgeries were performed by a trained veterinarian. Monkeys were pair-housed at all times, except when tick containment devices and jackets were in use; for that period, paired monkeys were in protected contact. Macaques received food (monkey chow) and water *ad libitum*, and standard enrichment (food supplements, manipulatable items in cage, human interaction with caretakers, perches or swings). Routine husbandry practices include the reporting of any abnormal clinical sign or activity by animals to the appropriate veterinary medical staff and faculty. Animal Care Technicians provided support during diagnostic and therapeutic procedures and the administration of the preventive medicine program. The standard method of euthanasia at the TNPRC, and that which was used, is anesthesia with ketamine hydrochloride (10 mg/kg) followed by an overdose with sodium pentobarbital. This method is consistent with the recommendation of the American Veterinary Medical Association guidelines.

Likewise, work with mice was in accordance with the Guide for the Care and Use of Laboratory Animals of the National Institutes of Health under the oversight of the University of Nevada, Reno Institutional Animal Care and Use Committee.

### Animals and Infection

**Figure 2:**
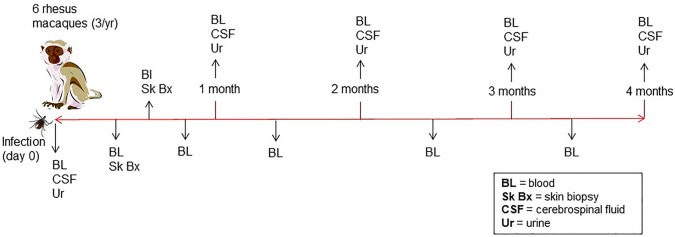
Time course of infection and sample collection—Rhesus macaques were infected with *B. burgdorferi* using a natural tick-bite model of Lyme disease. Blood, urine, and cerebrospinal fluid were collected throughout the 4-month infection.

A total of six male rhesus macaques (*Macaca mulatta*) of Indian origin were used to model human infection. The animals were inoculated with *B. burgdorferi* strain B31 by feeding infected ticks on them. This procedure has been optimized by the Embers lab and is described in a video publication (Embers et al., 2013). It was found that 50–90% of ticks feed to repletion with this method, ensuring transmission of the pathogen. The experimental protocol is shown in Figure 2. To confirm infection, 4 mm skin biopsies taken near the tick feeding sites were obtained at 1- and 2-weeks post-tick feed. Skin samples were subjected to culture and PCR, as described (Embers et al., 2012). From the blood collected at various time points (see below) serology was performed, using a recently-developed five-antigen test for *B. burgdorferi*-specific antibodies (Embers et al., 2016).

### Sample Collection

Blood, urine, and CSF were collected throughout a 4-month period following infection (Figure 2). A 4.9 ml tube of blood was collected at days 0, 7, and then every 2 weeks for the duration of the study. To preserve proteins in blood, protease inhibitors were introduced to the sample via the BD P100 system (Becton Dickinson) tubes used in collection. The tubes were centrifuged at 1900 × g for 10 min. to obtain serum. Urine and CSF were collected at day 0, 1 mo., 2 mo., 3 mo., and 4 mo. A protease inhibitor cocktail (cOmplete™, Mini, EDTA-free Protease Inhibitor Cocktail) was made into a 10x stock solution and was added immediately to each urine sample, for a final 1 x concentration, transported on ice and then stored at −80°C. At the end of 4 months, animals were euthanized and a gross necropsy was performed in order to obtain tissues in the event that the presence of *B. burgdorferi* needed to be verified.

### Acquisition of Host-Adapted *Borrelia burgdorferi* Proteins

The composition of antigens expressed by *B. burgdorferi in vitro* differs significantly from those expressed *in vivo*. Therefore, we utilized an *in vivo* culture system to acquire the proteins expressed by host-adapted spirochetes for analyses. The growth of *B. burgdorferi* strain B31.5A19 (Purser and Norris, 2000) in dialysis membrane chambers (DMCs) that were implanted into rat peritonea was performed as described previously (Akins et al., 1998). The initial quantity of organisms added to each bag was 5 ×10^5^/ml in a 5-ml volume. Rats were anesthetized by isoflurane gas (1.5 to 2% in oxygen) via nose cone through the entire procedure and received analgesics (buprenorphine subcutaneously at 0.1 mg/kg of body weight) postoperatively. Following implantation of DMCs and suture of rat incisions, organisms were grown for 14 days. Bacterial samples collected from each DMC were counted by dark-field microscopy and samples with the closest concentrations were pooled for processing. Protein lysates were prepared using two methods, protein extraction and sonication, with the purpose of including proteins that may have been diluted out using a single protocol.

For protein extraction, three DMCs were combined to give 1.6 ×10^7^/ml in a total volume of 13 ml. The spirochetes were pelleted (3,000 × g, 30 min., without brake), the supernatant was retained and the pellet was washed twice with PBS and resuspended in 1 ml of 50 mM Tris (pH 8)/10 mM EDTA/10% w/v sucrose. This was frozen in a dry ice/methanol bath (~3 min) and thawed in an ice water bath (~40 min). To this, 140 mM NaCl, 1 mM dithiothreitol (DTT) and 0.4 mg/ml lysozyme was added. This was incubated on ice for 45 min. with gentle mixing and subjected to 4 additional freeze/thaw cycles. Cell debris was removed by centrifugation at 18,000 × g in a fixed angle rotor. Samples were stored at −80°C in aliquots. This was also performed with *in vitro*-cultured *B. burgdorferi* (1 ×10^9^ cells total) (Embers et al., 2012).

To obtain sonicated preparations of *B. burgdorferi*, samples from individual DMCs with total quantities of spirochetes of 1.83 ×10^8^ and 1.36 ×10^8^ were pelleted and frozen for storage. Pellets were defrosted on ice, washed with 10 ml PBS and resuspended in 1 ml PBS on ice. The samples were sonicated with 8 pulses at amplitude 4 for 15 s each on ice. Samples were transferred to a microfuge tube and centrifuged for 5 min at 13,000 rpm to pellet debris. Protein concentrations were determined with a Nanodrop spectrophotometer (Thermo Fisher Scientific). Samples were stored in aliquots at −20°C.

### *In vivo* Microbial Antigen Discovery (InMAD)

BALB/c mice were immunized as previously described (Nuti et al., 2011). BALB/c mice were selected for the study as they have historically generated an array of antibodies, in high titers, in both InMAD studies as well as for the production of monoclonal antibodies. Briefly, the antigen is prepared by removal of whole microbial cells from the sample. For this experiment frozen serum samples from macaques KD91 and KC92, previously infected with *B. burgdorferi*, collected at 0, 1, and 2 weeks post-infection, were thawed, centrifuged at 10,000 rpm for 10 min followed by filtration through a 0.22 μm syringe filter to eliminate the mass of whole *B. burgdorferi* cells from the sample. Some cell lysis may have been induced through the removal of bacterial cells. The serum filtrates were then mixed 1:1 with TiterMax Gold Adjuvant and mixed in glass syringes to form an emulsion. Three mice (6–8 weeks old) were immunized via the subcutaneous route with 200 μl of each of the emulsion samples. Due to the limited volume of each sample of macaque serum, a boost of the immunization strategy was not included. Serum was collected from immunized mice, referred to InMAD immune serum, at 0, 4, 6, and 8-weeks post immunization via post retro-orbital bleed. The immune response generated by each mouse was monitored by assessing reactivity with *B. burgdorferi* whole cell lysates using a standard immunoblot. At 8 weeks post-immunization, the cardiac puncture method was utilized to obtain a final bleed from mice euthanized by extended isoflurane exposure.

### Protein Arrays

Initially, the antibody response generated by the infected macaques and immunized mice was gauged using protein array, contracted through Antigen Discovery (Irvine, Ca). Each array was printed with *in vitro* transcribed and translated open reading frames (orfs) supplemented with recombinant proteins, resulting in an array representing 1,397 proteins encoded for by *B. burgdorferi*. Serum from macaque KD91 collected at 6 weeks post-infection, and the pre-bleed and final bleed (8 weeks post-immunization) from a mouse immunized with serum from macaque KD91 2 weeks post-infection, were used to probe the array. Animal-specific IgG and IgM secondary antibodies were used to identify Ig type. Incubations with antibodies were 1 h at room temperature.

### Nucleic Acid-Programmable Protein Array (NAPPA)

NAPPA is a protein array technology that provides for on-array cell-free protein expression coupled with the capture and display of each protein in defined wells on the array surface. Antibodies found in a serum sample used to probe the array, highlight reactive proteins (Takulapalli et al., 2012). Each of 10 *B. burgdorferi* encoded genes (Table 1) included on the NAPPA were selected due to cellular localization. Genes were synthesized by ThermoFisher Scientific in the pENTR221vector and transferred into the pANT7_cGST destination vector. For plasmid preparation, the vectors were transformed into *E. coli* DH5α and purified by alkaline lysis. For printing, the plasmids were diluted into a Master Mix of printing components including bovine serum albumin, polyclonal anti-tag Ab (goat anti-GST) and a chemical cross-linker (BS-3). Positive controls on the array include Primate IgG and IgM (which confirms secondary reagent activity). Negative controls include empty parent plasmid pANT7_cGST, and Master Mix components without exogenous plasmid. The DNA/Master Mix contents of these 96 well plates are re-arrayed into 384 well plates which are then deposited onto aminosilane-coated silicon nanowell slides using a piezoelectric printing protocol (Bian et al., 2015; Song et al., 2017). Printed but unexpressed slides are stored under a dry argon atmosphere, as stability studies have shown that properly stored arrays generate comparable protein signals to freshly printed slides for >8 months after printing. Positive controls on the array include purified primate IgG and IgM, for confirming secondary antibody activity. Negative controls include empty parent plasmid pANT7_cGST (which only produces GST protein alone), and Master Mix components without exogenous plasmid.

**Table 1:** Genes or region included in the limited array.

**Gene**	**Accession number**
BB_A68	NP_045741.1
BB_A64	NP_045737.2
BB_A74	NP_045747.1
BB_K32	AAC66134
vlsE _C6 region	atg aag aag gat gat cag att gct gct gct att gct ttg agg ggg atg gct aag gat gga aag ttt gct gtg aag
BB_A15	NP_045688
BB_B19	NP_047005
BB_032	YP_008686571.1
BB_A24	NP_045697
BB_0147	NP_212281.1

Arrays were blocked with SuperBlock (Thermo Fisher Scientific) prior to expression to reduce non-specific binding, rinsed with DI water and centrifuged dry. The nano-wells were filled with human cell-free expression system reaction (*in vitro* Transcription and Translation coupled system; IVTT; Thermo Fisher Scientific) and a custom micro-reactor device was used for protein expression (Wiktor et al., 2015). After sealing the wells with a polystyrene membrane under pressure (200 PSI), the arrays were incubated for 2 h at 30°C for expression and for 0.5 h at 15°C for protein capture, and blocked for 30 min as above.

The nascent protein arrays were used for serum binding analysis using individual serum samples diluted 1:150 in 5% skim milk in PBS-T. Serum samples were derived from macaques infected with *B. burgdorferi* (see section Animals and Infection to Sample Collection). After overnight incubation (14–16 h) at 4°C with gentle shaking to ensure even exposure of array surface to sample, the arrays were rinsed and antibody binding was detected with AlexaFlour-647 labeled anti-primate or human IgG (H+L) and 1:200 diluted Cy3 labeled anti-primate or human IgM. The slides were rinsed again to remove unbound secondary antibody, dried by centrifugation and scanned at 635 nm and 535 nm with a Tecan PowerScanner. The resulting images were quantified with the ArrayPro Analyzer Software (Media Cybernetics, Inc.). Data was extracted and median normalized within each subarray. To assure a sufficient margin between positive and negative antibody reactivity we used a signal-to- noise ratio cutoff of 1.4 to identify spots for positive reactivity. This represents >3 standard deviations of the signals above the negative control samples and is a minimal signal-to-noise ratio known to provide detectable signals in ELISA validation assays (Song et al., 2017).

### Immunoprecipitation

All immunoprecipitation experiments were carried out using Dynabeads M-270 epoxy (Invitrogen). Antibodies were coupled to 5 mg of beads using the Dynabead antibody coupling kit. For coupling of purified antibodies, 50 μg of antibodies in PBS were coupled. For coupling of antibodies in serum, 150 μl of InMAD immune serum (8 weeks post-immunization) or infected macaque serum (6 and/or 8 weeks post-infection) was used. Antibody-coupled magnetic Dynabeads were used to pull down proteins in either macaque sera or protein lysates from *B. burgdorferi* adapted to host conditions (culture in DMC). Briefly, antibody-coupled beads were mixed with each sample (a final volume of 250 μl in a binding buffer [50 mM Tris-HCl, 1% Triton X-100, 1 mM EDTA, pH 7.6]) for 4–24 h rotating at 4°C and the beads were extracted from the solution using the Dynabead magnet. The beads were washed 4x with PBS. The captured antigens were eluted from the beads in 100 μl of 0.1 M citrate (pH 3.1) rotating 2 min at room temperature. The beads were separated out, and proteins in solution were transferred to a clean tube containing 20 μl neutralization buffer (1M Tris, pH 9). Eluted proteins were precipitated and digested for mass spectrometry or separated using SDS-PAGE. Due to limiting volumes of *in vivo* samples, the use of samples collected from independent macaques at distinct time points (e.g., 1- vs. 2-weeks post-infection) served as controls for immunoprecipitation studies. In that different proteins were identified from IP experiments from each sample, decreasing the likelihood that a protein was pull-downed through non-specific binding.

### Sample Preparation for Mass Spectrometry

Macaque sera isolated from each animal at 1- and 2- weeks post-infection and urine from 4 weeks post-infection were analyzed. CSF was not included in the analysis as it is a difficult sample to collect for diagnosis, however it is available for future studies. Samples were prepared for mass spectrometry using either the FASP method for sera or chloroform precipitation for urine samples, followed by trypsin digest (Yu et al., 2014). Prior to digestion, serum samples were depleted of the 14 most abundant proteins using the Hu-14 depletion column, per manufacturer's instructions (Agilent) and concentrated using a protein concentrator with a 10 kDa cut off. The Hu-14 column has been demonstrated to effectively remove abundant protein from macaque and canine samples (Barrett, 2004). Samples were prepared for analysis using in-solution digest with DTT, iodoacetamide, and trypsin. Immunoprecipitated proteins (see section Immunoprecipitation) were precipitated with a chloroform-methanol extraction prior to reduction, deacetylation, and digestion.

### Liquid Chromatography

The trypsin-digested peptides from each sample were analyzed by liquid chromatography-mass spectrometry using a discovery approach at the Nevada Proteomics Center (Univ. of Nevada, Reno). Briefly, peptide mixtures were separated using an UltiMate 3000 RSLCnano system (Thermo Fisher Scientific) on a self-packed UChrom C18 column and eluted using a digital Pico View nanospray source. Mass spectral analysis was performed using an Orbitrap Fusion mass spectrometer (Thermo FisherScientific). For analysis of results, tandem mass spectra were extracted and charge state deconvoluted by Proteome Discoverer version 2.1. All MS/MS samples were analyzed using Sequest and validated using Scaffold (version Scaffold_4.5.1) software. Peptide identification is reported as the X-correlation (cross-correlation value) as reported by the Sequest program.

## Results

### Infection Status and Serological Response to Exposure With *Borrelia burgdorferi*

**Figure 3:**
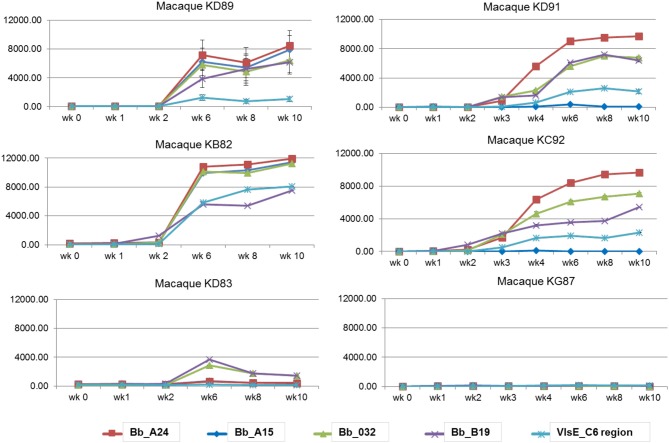
Serological response to a natural *B. burgdorferi* infection using a 5-antigen multiplex Luminex®-based assay. Each graph represents one animal, with the antigens detected distinguished by color. Note, only KD91, KC92, and KG87 were assessed at wks 2 and 3. Vertical axis: MFI, mean fluorescence intensity. Shown is the mean ±SEM for each time point. The mean values obtained from pre-immune serum of each individual macaque was subtracted from the MFI for each time point.

A macaque model of human infection was implemented to study the presence of microbial biomarkers in the host, as well as the host immune response to infection with the Lyme disease spirochete. This model was chosen because the disease process and variability in immune responses reflects those seen in humans. Skin biopsy was taken from each macaque near a tick bite site papule or patch of erythema. Analyses of the skin punches indicated that 5/6 macaques were biopsy-positive for *B. burgdorferi* (Table 2). The longitudinal serological response to OspC, OspA, DbpA, OppA2, and the C6 peptide of VlsE were assessed with a 5-antigen multiplex IgG assay as described (Embers et al., 2016) (Figure 3). Over the 6 week monitoring period 4/6 macaques developed an immune response that increased over time to at least 4 of the 5 antigens, and 5/6 macaques developed a response to 2 or more antigens. While 100% correlation was not seen between the biopsy and serological responses, the results indicate that all 6 animals initially developed an infection with *B. burgdorferi*.

**Table 2:** Clinical determinants of macaques infected using the tick-bite model of infection.

	**Skin Biopsy—PCR**	**Skin Biopsy—culture**	**Ticks removed (post-feeding)**
KD91	+	–	7
KC92	+	–	14
KG87	+	–	8
KB82	-	–	13
KB83	+	–	5
KD89	-	+	6 + 4 partial

*Including number of infected ticks removed from each macaque and infection status of the animals as determined by skin biopsy followed by PCR or culture*.

### Temporal Accumulation of Antibodies to *Borrelia*-Specific Antigens

**Figure 4:**
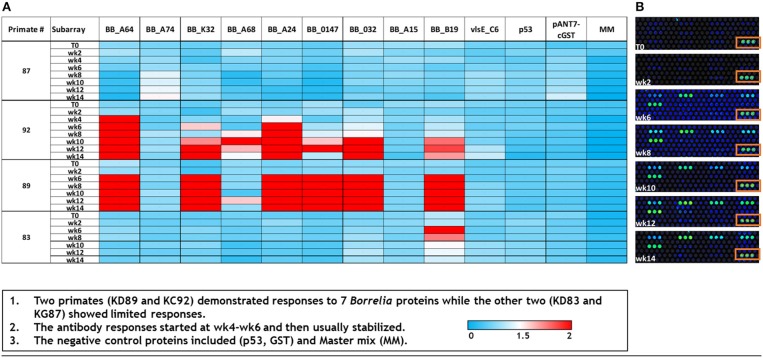
Dynamics of immunogenic response of macaques to *B. burgdorferi* as assessed by a limited NAPPA array. **(A)** Normalized signal intensities across the array were calculated by subtracting the background individual spot intensity of negative controls from the individual spot intensity. This is divided by the median array spot intensity minus the background spot intensity. Typically, a minimal signal-to-noise ratio of 1.4 is known to provide detectable signals in ELISA validation assays (Song et al., 2017). Serum used to probe the array was collected at 0–14 weeks post-infection (T0–T14). **(B)** A portion of the limited array representing the temporal response (week post-infection, T0–T14) of macaque KD89 to 6 *Bb* proteins, 5 negative controls, and 1 positive control (boxed). Each protein is represented by 3 spots on the array.

As a supplemental approach to assess the temporal pattern of antibody generation to *Borrelia* in macaques, a 10 protein *B. burgdorferi*-specific NAPPA array was developed. Protein selection (Table 1) for this limited array was based on protein localization (outer membrane) and interest as a diagnostic antigen. The array was probed with serum samples collected throughout the infection of macaques. The data indicate that a subset of the macaques developed an immune response to 7/10 *B. burgdorferi* proteins. The temporal pattern of the response was overlapping, but not constant between the animals (Figure 4). Importantly, a detectible response was not recorded from samples collected from macaque KG87 using either the 5-antigen multiplexed assay or the NAPPA (Figures 3, 4), indicating the animal remained seronegative throughout the sampling period. The infection status of this animal at the study end point was not evaluated. The variable pattern between macaques is a trend that is consistent with infection patterns in patients, as evident in the variability of the results from the current two-tier assay (i.e., 5 or more of the 10 proteins on the Western blot are required for a positive result) (Moore et al., 2016). The C6 reactivity by 4 of the 6 monkeys is apparent when the Luminex-based assay is performed, but was not detected but the NAPPA array. Perhaps this is due to the nature of the antigen—peptide vs. protein and how it is presented in each assay system. Please note that serum collected from a subset of the animals was used in this experiment, as samples from the complete time-course were not available from every animal.

### Direct Identification of Biomarkers

Sera and urine samples from infected macaques were submitted for analysis using a discovery approach to mass spectrometry. InMAD immune serum collected from mice was not submitted for mass spectrometry as microbial biomarkers at low concentrations in the macaque serum or urine would be diluted in the InMAD immune sera, further minimizing the chance of detection. Analyses of the serum samples resulted in identification of six antigens that met the criteria of potential biomarkers, two of which were detected late in infection by the 5-antigen multiplex serological assay; DbpA and VlsE (Table 3). In addition, two antigens, Fla and p83/100, defined as markers of the two-tier (immunoblot) assay were detected, suggesting that there is overlap between our strategy and established benchmarks (Dressler et al., 1993; Ryffel et al., 1998). Inclusion as a potential biomarker necessitated 2 or more identifications. Biomarkers identified through this small sample will be validated in a larger panel of macaque samples along with acute patient samples prior to inclusion in a diagnostic. While proteins specific to *B. burgdorferi* were identified from urine samples, they were only identified in single samples and therefore did not meet the criteria as potential target antigens.

**Table 3:** *Borrelia burgdorferi* antigens detected in infected macaque serum samples using a combination of mass spectrometry and protein array.

**High-potential biomarkers (3 or more identifications)**			
	Method	Sample	MS X-Correlation value or Array reactivity ranking (sample type)
DbpA (BB_A24)	Direct MS	Macaque KD91−1 and 2 week post infection	Range: 1.86–4.34
	Protein Array	Macaque KD91−6 week post infection	In top 25 IgG responses (macaque)
Fla (BB_0147)	IP/MS	InMad Immune sera KD91immunized (x3)—IP of *in vivo* lysates; KD89 T6 coupled-IP of *in vivo* lysates	Range: 1.83–5.90
	Direct MS	Macaque KD89−1week post-infection	2.79
	Protein Array	InMad immune sera−8 week post immunization	In top 25 IgM responses (mouse)
VIsE (BB_F0041)	IP/MS	Macaque KD89 antibodies coupled to beads—IP of macaque KD89 serum-2-week post-infection	2.36
	Direct MS	Macaque KG87−1 week post infection	2.62
	Protein Array	Macaque KD91−6 week post infection	In top 25 IgG responses (macaque) In top 35 IgM responses (macaque)
**Potential Biomarkers (2 identifications)**			
p83/100 (BB_0744)	Direct MS	Macaque KD91−1 and 2 weeks post-infection (pooled)	2.45
	Protein Array	InMad immune sera−8 weeks post immunizations	In top 25 IgM responses (mouse)
BB_G31	IP/MS	InMad immune sera coupled to beads—IP of *in vivo* lysate	2.19
	Direct MS	Macaque KD91−1 and 2 week post-infection (pooled)	2.51
BB_J48	Direct MS	Macaque KD91−1 and 2 week post-infection (not pooled)	Range: 2.22–2.35

*A range in X-correlation values reflects identification from multiple samples. X-correlation value is an indication of the alignment of the peptide detected with the predicted mass-to-charge ratio of the theoretical peptide as assessed by Sequest (cut-off for inclusion 1.8). The array reactivity ranking was a reflection of the florescent intensity on the array, sample type indicates if macaque sera or InMAD immune sera was used to probe the array*.

### Indirect Identification of Biomarkers

Proteins secreted or shed by *B. burgdorferi* in the serum or urine of the host may be at a concentration below the limit of detection by mass spectrometry. In order to enhance the prospect of detecting the proteins by mass spectrometry, immunoprecipitation was utilized to enrich samples for antigenic biomarkers. One key aspect of the InMAD process is that it allows for the generation of a diverse array of antibodies to biomarkers found early in infection. To increase the diversity of proteins that were isolated from infected animal samples and the host-adapted bacterial protein lysates (DMC-cultured), antibodies generated by mice in the InMAD immune sera, as well as by macaques at 6 weeks post-infection were used as receptors in immunoprecipitation experiments. By adding the immunoprecipitation step, an additional biomarker of interest was identified from a macaque sample that had already been detected by direct MS (VlsE; Table 3). In addition, proteins from host-adapted bacterial lysate (DMC-cultured) were enriched for immunogenic proteins, prior to identification by MS, resulting in the identification of two proteins of interest (Fla and BB_G31). It is important to note that DMC-cultured spirochetes are protected from the immune response, so antigens involved in immune evasion may be differentially expressed in this system.

### Identification of Antibodies Generated to *Borrelia burgdorferi*

A whole genome proteome array, produced and probed by Antigen Discovery, was utilized for initial studies, to detect immunogenic proteins in macaque and murine hosts. Each of the 1,397-*in vitro* transcribed and translated genes on the array were ranked by fluorescence intensity generated upon probing with each sample (Table S1). While data generated using the array is limited, using serum samples from a single macaque and one mouse from the InMAD experiment to probe the array, the data is included to support the mass spec results. As such, the data were considered as a single factor in our multi-platform approach to establish target antigens present in a model of *B. burgdorferi* infection (Table 3).

## Discussion

The gold standard tests for detection of many infectious diseases require that samples are sent to a central or specialty laboratory for culture and/or detection assay, processes that can take days to weeks for a definitive diagnosis. Furthermore, samples with a low bioburden may drive a false-negative-results without an amplification step, thereby adding additional time from sample collection to results. The CDC recommended assay for laboratory diagnosis of Lyme disease is a two-step serology-based assay, which requires the development of a prescribed immune response. The laboratory diagnosis is often considered secondary to patient history, including exposure of tick habitats. Disease diagnosis that is dependent upon the patient developing an immune response is problematic for multiple reasons, as (i) development of an antibody response can delay treatment by several weeks, (ii) the nature of seronegative patients would necessitate additional testing strategies, and (iii) serological assays do not distinguish between new and previously treated infections. These are among the reasons that a sensitive, defined antigen-based assay for early detection of many diseases is needed, with the inhibiting factor being the discovery of microbial biomarkers in patient samples that are within the level of detection of established assays. Minimal concentration of biomarkers early in infection may necessitate sample enrichment for successful biomarker discovery. Throughout the course of this study different enrichment strategies were used to identify microbial biomarkers, examples of enrichment are as follows (i) concentration of all biomarkers (concentration of host and microbial markers in urine), (ii) enrichment of microbial biomarkers (depletion of host proteins from serum), and (iii) enrichment of specific biomarker (immunoprecipitation). While each of these techniques may lead to loss of some targets, an experimental design that allows for data collection using overlapping approaches will minimize the loss. Beyond enrichment, the problem of low target concentration, as well as that of variation of biomarkers between patients, can be addressed through the inclusion of multiple microbial biomarkers in the development of a sensitive and specific diagnostic for early detection. The format of such multiplexed assays will be defined by the target limit of detection and adaptability to clinical workflow.

As a proof-of-principle, multiple platforms were exploited in an effort to unmask *B. burgdorferi* biomarkers that may have been missed in single step methods due to difference in concentrations of host vs. microbial proteins. By limiting samples used for both direct analysis and in the InMAD studies (which defines the immune response to circulating antigens at a specific point in time) to those isolated early in infection, only biomarkers that promote an early diagnosis should have been identified. A recent study by the Turko group, with a similar goal of identifying biomarkers for Lyme disease, focused on identifying biomarkers found to be abundant in *B. burgdorferi* B31 cultured *in vitro*, in patient samples using MS. Their studies found that peptides from the OspA could be detected in early patient serum samples upon concentration, but not in samples collected later in infection (Cheung et al., 2015). OspA, a potential biomarker that was not highlighted in our study was also found in urine samples concentrated by Nanotrap (Magni et al., 2015). These reports confirm the idea presented here that in order detect low-abundance *B. burgdorferi* proteins, sample concentration is critical.

Samples used in this study were based upon a well-defined model of disease that closely mimics Lyme disease in humans, namely the macaque model of infection by tick vector, which was combined with the temporally defined InMAD assay (Nuti et al., 2011; Caskey and Embers, 2015; Crossland et al., 2018). A conservative approach to biomarker identification was taken by defining hypothetical target antigens as those that were identified using more than a single technique or in multiple samples. More specifically, proteins that were identified more than once were classified as potential biomarkers, and those identified three or more times were classified as high-potential biomarkers. The data generated using the platform was integrated to identify six proteins that were detected as candidate early microbial indicators of infection. Target biomarkers present in serum from the infected host included both targets previously discussed as diagnostic antigens as well as those that are not normally considered candidates for use as a diagnostic of Lyme disease, opening new avenues of research. Furthermore, more emphasis was placed on serum than urine samples, allowing for the possibility that additional microbial biomarkers may be present in urine. Candidate early microbial biomarkers for Lyme are undergoing further validation as part of the development of an early diagnostic. And the multi-platform approach to biomarker discovery, defined in this study, is currently being used to define antigenic biomarkers for *Francisella tularensis* and *Burkholderia pseudomallei*.

## Data Availability

All datasets generated for this study are included in the manuscript and/or the Supplementary Files.

## Ethics Statement

Practices in the housing and care of non-human primates and rodents conformed to the regulations and standards of the Public Health Service Policy on Humane Care and Use of Laboratory Animals, and the Guide for the Care and Use of Laboratory Animals. The Institutional Animal Care and Use Committees of Tulane University and the University of Nevada, Reno approved all animal-related protocols.

## Author Contributions

KP, DM, JL, MP, ME, and DA designed the experiments and wrote the manuscript. KP, MM, NH, MJ, AT, DM, LS, and ME conducted the experiments. All authors read the manuscript.

### Conflict of Interest Statement

DA, KP, and MM are current employees of DxDiscovery Inc. The remaining authors declare that the research was conducted in the absence of any commercial or financial relationships that could be construed as a potential conflict of interest.

## Abbreviations

InMAD: *in vivo* Microbial Antigen Discovery
NAPPA: Nucleic Acid-Programmable Protein Array.
