# Supplementary material for: Multi-platform Approach for Microbial Biomarker Identification Using Borrelia burgdorferi as a Model
Source: Front Cell Infect Microbiol. 2019 Jun 11;9:179. doi: 10.3389/fcimb.2019.00179 (PMC6579940; doi:10.3389/fcimb.2019.00179)
Supplement: Supplementary file 1 [file Table_1.pdf]

Table S1: Protein array reactivity with IgG or IgM in InMAD immune sera (Mouse) or Macaque Sera

ID	Mouse.Prebleed.IgG	Mouse.Final.IgG	Macaque.IgG	Mouse.Prebleed.IgM	Mouse.Final.IgM	Macaque.IgM
BB_S41	0.138	0.264	3.616	0.573	0.766	1.956
BB_0836 (fcqc)	-0.106	0.037	3.616	0.266	0.646	1.058
BB_K50-m2	0.266	0.353	3.615	0.114	0.379	3.277
BB_K07	0.349	0.315	3.615	0.707	0.954	1.759
BB_L39-m2	0.566	0.861	3.615	0.167	0.251	2.047
OspC_B31	0.298	0.161	3.615	0.181	0.504	2.502
BB_B19-m1	0.344	0.406	3.615	0.293	0.463	2.068
BB_A04	0.296	0.727	3.615	0.467	0.682	1.263
BB_A36	0.553	0.643	3.615	0.283	0.62	1.096
BB_R42-m2	0.5	0.41	3.615	-0.028	0.022	0.372
BB_A24-noss	0.271	0.189	3.615	-0.053	-0.049	0.411
BB_K12	-0.002	0.107	3.614	0.391	0.795	1.447
BB_A52-m1	0.354	0.358	3.614	0.087	0.184	0.794
BB_M27	0.425	0.68	3.61	0.431	0.591	0.709
BB_B19-m2	0.421	0.454	3.609	0.2	0.36	2.228
BB_O39	-0.145	-0.044	3.598	0.191	0.392	0.872
BB_K47	-0.053	0.071	3.589	-0.024	0.103	1.52
BB_K49	-0.191	-0.185	3.517	0.058	0.183	1.294
BB_R42-m1	0.189	0.12	3.506	0.005	0.193	0.471
BB_L40	0.191	0.354	3.474	1.052	0.983	1.469
vlsE nt56-1069	0.124	-0.107	3.46	0.132	0.085	1.327
BB_0289	0.54	0.567	3.438	0.741	1.01	0.644
BB_A25-m2	0.566	0.832	3.415	0.061	0.066	0.587
BB_A57	0.185	0.1	3.4	1.483	1.839	1.276
OspC_N40	0.571	0.488	3.396	0.512	0.83	0.991
BB_N39	-0.212	-0.075	3.381	0.037	0.09	0.652
BB_K19	0.484	0.512	3.374	0.555	1.166	0.723
BB_S41 noss stB31	-0.111	-0.115	3.354	0.338	0.377	0.888
BB_F32#2 (fcqc)	-0.271	-0.238	3.303	0.013	-0.04	0.717
BB_0282	0.869	0.802	3.204	0.184	0.499	0.763
BB_F01	0.754	0.711	3.184	0.975	1.83	1.228
OspC_Type6	0.574	0.391	3.177	0.292	0.849	0.827
BB_K50-m1	-0.265	-0.214	3.138	0.04	0.12	3.319
BB_A66	0.353	0.619	3.092	0.101	0.273	2.764
OspC_Type5	0.431	0.378	3.09	0.199	0.293	0.797
BB_0844	-0.482	-0.247	3.06	0.1	0.009	0.151
BB_N38	0.132	0.185	3.058	-0.013	0.047	1.383
BB_A25-m1	0.318	0.377	2.973	0.308	0.345	0.89
vlsE nt12-1069	-0.181	-0.257	2.948	-0.02	-0.096	0.307
BB_O40-m1	0.132	0.205	2.881	0.049	0.061	0.131
BB_C10	-0.103	0.091	2.821	0.015	0.043	2.068
BB_O40-m2	0.058	0.041	2.814	-0.047	-0.013	-0.102
BB_0383-noss	0.433	0.458	2.796	0.01	0.081	0.5
BB_A07	0.061	0.144	2.725	0.018	0.11	0.263
BB_0147	0.103	0.225	2.679	0.543	1.13	1.754
BB_K48	0.235	0.411	2.651	0.494	0.659	2.402
BB_J47	1.08	0.997	2.645	0.154	0.305	0.18
BB_G18	0.438	0.498	2.621	-0.032	0.049	-0.033
BB_0710	-0.026	0.089	2.606	-0.087	0.01	0.082
BB_0279	0.343	0.288	2.549	1.606	2.119	0.794
BB_M38-m2	0.288	0.258	2.505	0.189	0.313	2.154
BB_0203	0.222	0.134	2.5	0.136	-0.01	-0.092
BB_F32#1	0.882	0.787	2.444	0.044	0.125	0.624
BB_0744-noss	-0.675	-0.674	2.442	0.29	0.996	0.602
BB_N28	0.671	0.493	2.412	0.131	0.617	0.821
BB_P39	-0.402	-0.183	2.407	-0.115	0.02	0.144
AF213178 st IP90	0.591	0.695	2.367	0.217	0.178	0.261

OspC_Type4	0.741	0.844	2.245	0.33	0.515	0.795
X75203 noss st IP90	0.492	0.336	2.229	0.307	0.637	1.315
BB_A03-m2	0.505	0.781	2.159	0.719	0.424	1.094
BB_0749	0.418	0.319	2.128	0.613	0.578	1.655
BB_M38-m1	0.049	0.094	2.122	0.074	0.032	1.645
BB_0268	1.011	1.102	2.104	0.612	1.03	1.537
BB_A64	0.473	0.548	2.072	0.748	0.284	1.464
ETEC_1674	-0.014	0.929	2.051	0.12	0.158	0.423
BB_L18	-0.039	-0.063	2.027	0.092	0.116	0.181
BB_J34	0.737	0.621	2.025	0.189	0.314	0.74
BB_0286	0.46	0.528	2.003	0.062	0.695	1.77
BB_J36	0.695	0.596	1.992	0.068	0.263	0.313
BB_0543	0.69	0.667	1.978	0.028	0.183	1.057
ETEC_1771 1_4	0.418	1.201	1.969	0.12	0.101	0.131
BB_J50-m1	0.366	0.406	1.908	0.096	0.186	0.971
BB_A55 . duplicate	0.119	-0.006	1.884	0.128	0.097	-0.105
BB_0210	0.731	0.814	1.847	0.641	0.672	0.604
ETEC_1771	-0.228	0.998	1.828	0.062	0.056	0.103
BB_A62-m1	0.973	0.28	1.818	0.088	0.26	0.929
BB_0344	0.21	0.508	1.791	0.195	0.3	0.581
ETEC_1674 1_4	-0.399	0.562	1.733	0.008	-0.026	0.027
BB_O16	0.4	0.393	1.727	0.146	0.145	0.252
OspC_HB19	0.989	0.89	1.698	0.269	0.605	0.747
BB_H18	0.135	0.139	1.663	-0.046	-0.028	-0.287
BB_I42	0.898	0.687	1.625	0.197	0.218	1.031
BB_0348	0.098	0.219	1.623	0.055	0.269	0.408
BB_R29	-0.212	-0.091	1.623	0.028	0.264	0.413
BB_K32	0.09	0.117	1.589	0.039	0.191	0.07
BB_A72	-0.096	0.1	1.576	0.233	0.04	0.039
BB_E31. duplicate	0.75	0.774	1.534	-0.008	0.363	0.399
BB_H39 (fcqc)	0.105	-0.005	1.509	-0.042	-0.157	-0.177
BB_R02	0.091	-0.098	1.5	0.466	0.236	0.197
BB_Q52	-0.282	-0.324	1.493	1.547	0.694	0.642
BB_Q35-m2	-0.072	0.009	1.471	0.013	0.167	0.545
BB_A03-m1	0.455	0.907	1.463	0.851	0.627	1.443
BB_O02	-0.664	-0.501	1.448	1.492	0.724	0.235
BB_I22 (fcqc)	0.201	-0.123	1.437	0.026	0.261	0.007
BB_J09	0.51	0.523	1.418	0.165	0.195	1.45
BB_0096	0.408	0.641	1.413	0.478	0.774	0.686
BB_N02	-0.608	-0.433	1.403	1.242	0.49	0.429
BB_C06-m2	0.27	0.798	1.354	0.479	0.602	0.81
BB_Q34	0.212	0.25	1.352	0.181	0.249	0.448
BB_0108	0.047	0.24	1.352	0.069	0.05	0.324
BB_N38-1 st. HB19 noss	0.098	0.097	1.317	-0.035	0.044	0.397
BB_L02	-0.273	-0.121	1.31	1.043	0.389	0.096
BB_R28-m2	0.451	0.386	1.27	0.233	0.268	2.648
BB_0603	0.953	0.854	1.247	0.105	0.28	0.843
BB_0296	0.543	0.683	1.236	0.316	0.563	0.667
BB_0512	0.526	0.575	1.228	0.198	0.472	0.69
BB_0664	0.987	0.954	1.178	0.651	0.874	2.804
BB_B09	-0.062	-0.212	1.16	-0.123	0.05	0.272
BB_E28 (fcqc)	0.652	0.608	1.154	0.071	0.163	0.868
BB_0783 (fcqc)	0.164	0.296	1.137	0.093	0.16	0.388
BB_0415	-0.389	-0.303	1.131	-0.067	0.056	-0.215
BB_H13	0.339	0.384	1.101	0.281	0.701	0.474
BB_A48	0.105	0.081	1.09	0.467	0.66	0.757
BB_O15	-0.06	0.385	1.087	-0.023	0.097	0.083
BB_L28-m1	1.126	1.092	1.08	0.121	0.542	1.07
BB_A70	-0.051	0.074	1.079	0.88	1.013	0.32

BB_R35	-0.648	-0.496	1.076	-0.11	-0.114	-0.107
BB_L38	0.377	0.787	1.056	0.867	0.937	-0.144
BB_A50	-0.153	-0.21	1.034	-0.168	-0.151	-0.277
BB_N38-2 st. HB19 noss	0.189	0.027	1.012	0.005	-0.001	0.205
BB_K53	0.184	0.437	1.009	0.095	0.306	0.88
BB_I06	0.15	0.043	0.984	0.053	0.14	0.047
BB_Q40	-0.298	-0.28	0.972	-0.084	-0.022	0.188
BB_0114	0.46	0.358	0.956	0.129	0.169	0.367
BB_A69-m2	0.348	0.273	0.954	0.123	0.18	-0.128
BB_P34	0.194	0.343	0.929	0.906	1.553	0.715
BB_H37	0.545	0.475	0.924	0.298	0.351	0.454
BB_0559	0.347	0.343	0.919	0.179	0.381	0.218
BB_M28	0.759	0.61	0.918	0.135	0.608	2.562
OspC_IP90	0.062	0.11	0.908	0.375	0.593	0.481
BB_0337	0.204	0.298	0.892	0.019	-0.127	0.002
BB_N34	0.22	0.21	0.889	0.434	0.298	0.173
BB_0506	0.889	0.929	0.875	0.179	0.422	0.664
BB_0312	0.686	0.688	0.86	1.802	1.97	0.834
BB_Q03	0.478	0.501	0.85	0.143	0.219	0.956
BB_H06 . duplicate	0.081	0.122	0.849	0.337	0.499	0.839
BB_K05	1.299	0.885	0.808	0.227	0.347	1.353
BB_A59-m1	0.489	0.527	0.799	0.511	0.889	0.705
BB_0713	-0.094	0.115	0.787	0.059	-0.009	-0.74
BB_0148	0.969	0.742	0.782	0.228	0.588	1.481
BB_F03	0.43	0.522	0.781	1.46	1.467	1.907
blank	0.744	0.691	0.775	-0.031	0.138	2.087
BB_0655	0.26	0.186	0.754	0.593	0.485	0.423
BB_0581 (fcqc)	0.244	0.204	0.742	0.606	0.689	0.429
BB_O34	-0.125	0.11	0.74	0.279	1.067	0.749
BB_0439	-0.055	-0.053	0.736	0.499	0.47	0.668
BB_H31	0.492	0.483	0.732	0.01	0.085	-0.057
BB_0839	0.3	1.143	0.731	0.231	0.125	0.686
BB_I34	0.679	0.741	0.727	0.697	0.498	0.919
BB_O28	0.592	0.6	0.717	0.037	0.587	1.445
BB_S30-m1	0.027	0.256	0.684	0.327	0.309	0.764
BB_0553	-0.234	-0.188	0.68	0.046	0.088	-0.071
BB_F10	0.098	0.118	0.673	0.106	0.111	2.051
BB_K46	0.819	0.723	0.671	0.816	0.598	2.827
BB_A13	0.399	0.444	0.665	0.126	0.048	1.718
BB_A16	-0.016	-0.013	0.657	-0.098	-0.073	0.307
BB_0065 (fcqc)	0.379	0.25	0.647	0.61	0.606	0.521
BB_Q54	0.731	0.589	0.641	0.211	0.326	0.022
BB_0037	0.047	-0.04	0.639	0.374	0.354	0.532
BB_G27	0.434	0.86	0.638	0.559	0.352	0.482
BB_J19	0.009	0.197	0.636	0.327	0.481	0.884
BB_F13	0.532	0.402	0.631	0.123	0.02	-0.156
BB_A74	0.573	0.559	0.62	0.637	0.804	0.612
BB_N38 st. 297 noss (fcqc)	-0.014	-0.018	0.62	0.459	0.484	0.277
BB_N26	0.722	0.796	0.619	0.342	0.374	0.273
BB_K13	0.577	0.674	0.607	-0.011	0.339	0.703
BB_L31	0.69	0.803	0.606	0.366	0.525	0.814
BB_S30-m2	0.521	0.502	0.6	0.045	0.625	2.218
BB_R28-m1	0.397	0.189	0.595	-0.033	0.001	1.629
BB_K24	0.466	0.317	0.591	-0.113	-0.138	-0.754
BB_0158	0.487	0.506	0.588	0.107	0.212	0.541
BB_0194	-0.123	0.1	0.588	0.553	0.6	0.444
BB_G33	0.489	0.643	0.585	0.339	0.442	0.583
BB_F25	0.654	0.47	0.585	-0.096	0.396	0.522
BB_A59-m2	0.586	0.533	0.583	0.094	0.036	-0.012

BB_L33	0.446	0.502	0.582	0.12	0.142	1.267
BB_0365	0.505	0.385	0.579	0.076	0.129	0.366
OspC_297	0.68	1.143	0.577	0.573	0.829	0.709
BB_0338	0.281	0.128	0.572	-0.047	0.051	0.029
BB_L27	0.201	0.33	0.566	0.513	0.709	0.829
BB_0602	-0.163	-0.016	0.562	0.421	0.302	0.176
BB_0684	0.456	0.31	0.555	-0.035	0.044	-0.178
BB_N18. duplicate	-0.352	-0.011	0.553	0.222	0.237	0.219
OspC_Type8 (fcqc)	0.356	0.347	0.551	0.181	0.64	0.312
BB_Q07	0.017	0.264	0.55	0.088	0.234	0.248
BB_0519	0.46	0.385	0.542	0.453	0.692	1.276
BB_B07-m1	0.229	0.287	0.541	0.085	0.179	0.364
BB_0310	0.218	0.17	0.539	0.024	0.04	0.21
BB_H06. duplicate	0.195	0.13	0.533	-0.05	-0.023	-0.337
BB_A64 noss st B31 (fcqc)	0.04	0.522	0.531	0.33	0.309	0.65
BB_K52 (fcqc)	0.1	0.042	0.531	0.037	0.202	0.704
BB_0563	1.164	1.148	0.527	0.331	0.276	0.496
BB_D21 . duplicate	-0.28	-0.068	0.524	0.118	0.354	0.24
BB_S21	0.479	1.477	0.52	0.226	0.337	0.508
BB_0377	0.787	0.176	0.52	0.316	0.184	0.187
BB_J09 noss st 297	-0.22	-0.082	0.52	-0.003	0.085	0.74
BB_0567	-0.163	-0.162	0.511	2.16	1.085	1.534
BB_Q41	-0.077	0.036	0.511	-0.105	-0.009	0.155
BB_I08.1	1.252	0.775	0.505	0.392	0.524	0.818
BB_0054	1.171	1.085	0.504	2.133	2.83	1.869
BB_0209	0.534	0.497	0.503	0.001	0.38	0.206
BB_0251	0.277	0.174	0.501	0.042	0.188	0.152
BB_0800	0.171	0.101	0.5	0.894	0.619	0.022
BB_0265	0.792	0.957	0.497	0.428	0.5	1.117
BB_A20	-0.524	-0.416	0.494	-0.079	-0.057	0.186
BB_0663	0.677	0.82	0.492	0.46	0.651	1.022
BB_0796	0.552	0.528	0.491	-0.103	-0.055	0.063
BB_0220	-0.268	-0.185	0.49	-0.101	-0.17	-0.342
BB_E09	1.134	0.638	0.488	0.364	0.601	0.83
BB_0445	0.327	0.503	0.484	0.247	0.569	0.682
BB_0484	0.11	0.115	0.481	0.025	0.016	0.467
BB_S31	0.455	0.411	0.48	0.194	0.259	0.442
BB_0698	0.525	0.598	0.472	0.163	0.29	0.985
BB_Q48	0.393	0.563	0.471	0.214	0.334	0.723
BB_I10	-0.414	-0.154	0.463	0.202	0.292	0.272
BB_N27	0.473	0.46	0.459	0.439	0.543	0.376
BB_0673	0.589	0.585	0.455	0.328	0.634	1.043
BB_K41	-0.065	-0.055	0.452	0.007	-0.025	1.228
BB_0280	0.787	0.781	0.444	0.095	0.368	0.544
vlsE nt731-1105 (fcqc)	0.719	0.745	0.441	0.107	0.197	1.578
BB_G15	1.049	0.985	0.44	0.128	0.26	0.329
BB_0629	0.171	0.086	0.438	-0.035	0.128	-0.092
BB_0724	1.222	0.919	0.437	0.115	0.244	0.595
BB_0352 (fcqc)	0.197	0.222	0.436	0.012	0.11	0.496
BB_0513	0.798	0.844	0.435	0.079	0.262	0.392
BB_O17	0.387	0.706	0.432	0.273	0.344	0.812
BB_N15	0.028	-0.217	0.432	-0.071	-0.085	-0.138
BB_0341	0.878	0.914	0.431	0.027	0.511	0.108
BB_0832	0.598	0.573	0.43	0.268	0.518	0.813
BB_C02	1.384	0.611	0.426	0.27	0.479	0.651
BB_0694	0.22	0.208	0.426	0.065	0.129	0.43
BB_0277	-0.12	0.002	0.425	-0.051	0.045	0.021
BB_0534	0.521	0.302	0.419	0.081	0.199	0.802
BB_0831	0.767	0.695	0.418	0.073	0.217	0.247

BB_A75	0.251	0.308	0.418	0.169	0.211	0.874
BB_B17	0.121	-0.004	0.415	0.102	0.131	0.304
BB_0535	0.69	0.662	0.405	0.331	0.562	0.825
BB_0418	0.626	0.755	0.405	0.243	0.614	0.768
BB_M34	0.212	-0.128	0.402	0.248	0.441	0.593
BB_0649	0.339	0.004	0.401	0.469	1.274	0.882
BB_B14	0.275	0.355	0.4	0.109	0.344	0.75
BB_0742 (fcqc)	-0.268	0.009	0.4	-0.046	0.042	0.475
BB_0278	0.518	0.39	0.397	0.239	0.366	0.533
BB_0233	0.775	0.948	0.394	0.169	0.585	0.674
BB_0163	0.327	0.343	0.393	0.245	0.514	0.553
BB_J39.1 (fcqc)	-0.139	-0.101	0.393	-0.072	-0.055	-0.083
OspC_ADDIE (fcqc)	0.399	0.293	0.392	0.352	0.653	0.943
BB_A05-m2	0.987	1.054	0.388	0.01	0.207	0.498
BB_0107	0.499	0.502	0.388	0.12	0.185	0.402
BB_0472	0.197	0.382	0.388	0.075	0.341	0.675
BB_0005	0.157	0.094	0.387	0.056	0.083	0.612
BB_0443	0.505	0.548	0.384	0.211	0.274	0.576
BB_0517 (fcqc)	0.626	0.824	0.382	0.111	0.281	0.504
BB_0670	0.101	0.531	0.381	0.254	0.634	1.958
BB_J09 noss st HB19	-0.107	0.036	0.38	0.073	0.163	0.576
BB_0475	0.699	0.728	0.378	0.352	0.603	0.858
BB_0468	0.994	0.643	0.375	0.169	0.459	0.63
BB_0390	-0.12	-0.071	0.374	0.148	0.142	0.073
BB_T04	0.578	1.21	0.373	0.143	0.509	0.65
BB_L28-m2	0.663	0.58	0.372	-0.055	-0.103	0.169
BB_0230	-0.16	-0.094	0.371	0.198	0.352	-0.281
BB_0573	0.826	0.581	0.369	0.314	0.512	0.743
BB_B21	0.689	0.702	0.368	-0.001	0.033	0.434
BB_A27	0.685	0.494	0.366	0.154	0.188	0.545
BB_0215	0.572	0.59	0.366	-0.026	0.025	0.498
BB_G13	0.557	0.551	0.364	0.184	0.436	0.786
BB_Q74	-0.108	-0.134	0.364	0.362	0.369	0.088
BB_Q06	1.008	0.778	0.363	0.226	0.266	0.518
BB_0790	0.813	0.747	0.361	0.195	0.187	0.422
BB_O24	0.253	0.26	0.361	-0.057	0.004	0.566
BB_0008	0.425	0.341	0.36	-0.043	0.084	0.033
BB_C06-m1	0.083	-0.035	0.358	-0.031	-0.067	-0.098
BB_0458	0.524	0.474	0.357	0.321	0.527	0.769
BB_0070	0.06	0.326	0.355	-0.096	0.019	0.135
BB_N37#2	0.499	0.705	0.354	-0.038	0.303	0.638
BB_0066	0.75	1.181	0.353	0.178	0.294	0.717
BB_0406	0.779	0.83	0.352	0.032	0.131	0.146
BB_0024 (fcqc)	0.679	0.875	0.35	0.174	0.696	0.927
BB_0478	0.303	0.273	0.35	0.07	0.299	0.64
BB_G31	0.283	0.28	0.349	0.127	0.164	0.812
BB_0234	1.095	0.882	0.348	0.21	0.32	0.716
BB_H10.1	0.366	0.295	0.346	0.026	0.102	0.815
BB_0307	1.423	1.396	0.345	0.114	0.103	0.184
BB_0431	0.196	0.302	0.34	0.023	0.235	1.843
BB_0791	0.43	0.595	0.34	0.245	0.417	0.618
BB_0271 (fcqc)	0.027	0.305	0.34	0.015	0.242	0.567
BB_0427	0.681	0.786	0.338	0.231	0.239	0.489
BB_0378	0.772	0.67	0.338	0.125	0.172	0.477
BB_D16	0.255	0.31	0.337	0.183	0.21	0.339
BB_R12	0.486	0.533	0.335	0.077	0.29	0.51
BB_A18	0.292	0.274	0.335	0.176	0.382	0.639
BB_0757	0.133	0.264	0.333	-0.005	0.099	0.173
BB_0330	0.148	-0.061	0.332	0.18	0.12	0.011

BB_G25	0.867	0.753	0.329	0.156	0.28	0.463
BB_K39	0.191	0.27	0.329	0.101	0.177	0.3
BB_0235	0.849	1.005	0.328	0.197	0.265	0.686
BB_0399	0.554	0.583	0.328	-0.077	0.044	-0.005
BB_0565	0.341	0.414	0.327	0.235	0.362	1.099
BB_I20	0.453	0.989	0.327	0.147	-0.299	-0.537
BB_A46	0.186	0.618	0.326	0.067	0.165	0.192
BB_G05	0.448	0.781	0.325	0.277	0.464	0.364
BB_0126	0.543	0.643	0.325	-0.069	0.013	0.103
BB_0620 (fcqc)	0.042	0.224	0.324	0.165	0.158	0.968
BB_0814	1.016	0.999	0.322	0.119	0.515	0.66
BB_0588	0.963	0.869	0.321	0.278	0.318	0.469
BB_0599	0.16	0.282	0.32	0.129	0.281	0.785
BB_A73	0.388	0.453	0.319	0.04	0.3	0.644
BB_0361	0.984	0.838	0.318	0.034	0.236	1.165
BB_B12	-0.032	0.041	0.318	-0.028	-0.094	0.09
BB_G28	0.198	0.462	0.317	2.059	2.268	1.304
BB_0223	0.307	0.293	0.316	0.112	0.201	0.656
BB_J40	-0.066	0.027	0.316	-0.072	0.038	0.067
BB_0554	0.132	0.423	0.315	0.083	0.437	0.718
BB_0815	0.288	0.609	0.315	0.151	0.108	0.234
BB_0028	0.203	0.241	0.312	0.14	0.378	0.925
BB_0142	0.659	0.643	0.31	0.37	0.563	0.623
BB_0073	0.923	0.905	0.309	0.157	0.335	0.514
BB_R31	0.446	0.629	0.309	0.248	0.588	0.595
BB_N18 . duplicate	1.182	1.136	0.308	0.133	0.263	1.605
BB_0140	0.979	0.879	0.308	0.177	0.154	0.492
BB_0306 . duplicate	0.686	0.643	0.308	0.075	0.13	0.187
BB_0451	0.722	0.798	0.307	0.05	0.225	0.461
BB_E21	0.14	0.28	0.307	0.206	0.629	0.109
BB_0359	0.567	0.565	0.305	-0.011	0.057	-0.119
BB_0589	0.88	0.7	0.304	0.158	0.372	0.634
BB_0682	0.424	0.439	0.304	0.211	0.349	0.6
BB_0360	1.107	1.148	0.303	0.137	0.203	0.437
BB_0564	0.737	0.897	0.302	0.02	0.231	2.786
BB_B27	0.659	0.78	0.302	0.304	0.515	0.544
BB_E12	1.256	1.135	0.3	0.08	0.115	0.627
BB_0507	0.551	0.568	0.298	0.275	0.661	0.301
BB_0688	0.518	0.52	0.295	0.108	0.21	0.294
BB_B28	-0.521	-0.457	0.295	0.285	0.454	0.155
BB_S27	1.263	1.196	0.293	0.501	0.96	0.785
BB_0811	0.163	0.724	0.292	0.045	0.254	0.416
BB_0792	0.664	0.801	0.29	0.164	0.286	0.397
blank	0.42	0.516	0.29	0.158	0.214	0.658
BB_G24	0.744	0.495	0.289	0.055	0.125	1.034
BB_0798 (fcqc)	0.195	0.157	0.289	-0.082	-0.019	-0.15
BB_0522	1.011	0.974	0.287	0.193	0.292	0.295
BB_R34	0.575	0.429	0.287	0.098	0.245	0.722
BB_0584	0.478	0.367	0.287	-0.019	0.098	0.043
BB_K20 (fcqc)	-0.263	-0.136	0.287	-0.078	-0.041	-0.048
BB_0034	1.08	1.184	0.286	0.308	0.316	0.474
BB_S40	0.488	0.538	0.286	-0.083	0.522	0.63
BB_G03. duplicate	1.263	1.11	0.285	0.119	0.167	0.861
BB_0083	0.409	0.505	0.285	0.728	0.95	0.923
BB_0532	0.849	0.622	0.284	0.076	0.091	0.155
BB_H05	0.283	0.526	0.284	0.164	0.259	0.557
BB_J48	0.529	0.808	0.284	-0.02	0.157	0.276
BB_0328	0.042	-0.024	0.284	0.041	0.153	0.494
BB_0409	1.186	1.222	0.281	0.435	0.467	0.65

BB_Q22	-0.659	-0.666	0.281	-0.089	-0.129	-0.201
BB_0079	0.483	0.956	0.28	-0.001	0.045	0.1
BB_A11	0.225	0.262	0.279	0.071	0.26	0.209
BB_K43	-0.374	-0.127	0.277	-0.134	-0.095	0.069
BB_0239	1.02	0.922	0.276	0.077	0.282	0.588
BB_0162	-0.224	-0.191	0.275	0.001	0.02	0.181
BB_0635	0.716	0.769	0.274	0.055	0.211	0.538
BB_0764	0.345	0.259	0.274	0.026	0.104	0.969
BB_0555	0.103	0.235	0.274	0.046	0.121	0.534
BB_O35	0.014	0.091	0.274	0.055	0.094	0.288
BB_0144	0.247	0.307	0.273	0.131	0.19	0.312
BB_0704	0.265	0.312	0.272	0.393	0.261	0.379
BB_0769	0.238	0.317	0.272	-0.027	0.094	0.547
BB_0010	0.987	0.96	0.27	0.094	0.239	0.683
BB_0745	0.825	0.69	0.27	0.105	0.25	0.146
BB_0023	0.427	0.647	0.268	0.245	0.408	0.839
BB_N31	0.492	0.603	0.267	0.183	0.437	0.617
BB_0071	0.329	0.552	0.267	-0.007	0.243	0.277
BB_0347	0.551	0.446	0.266	0.133	0.461	0.712
BB_A19	0.161	0.603	0.266	0.155	0.17	0.319
BB_I13	-0.027	-0.25	0.266	-0.023	-0.065	-0.157
BB_0334	0.09	0.054	0.264	0.066	0.145	0.479
BB_0062	1.481	0.621	0.263	0.286	0.55	0.129
BB_0119 (fcqc)	-0.12	-0.172	0.263	-0.067	-0.015	-0.049
BB_0161	0.427	0.401	0.262	0.124	0.549	0.718
BB_0004	0.212	-0.109	0.261	-0.039	0.118	0.452
BB_0648 (fcqc)	0.157	0.269	0.261	-0.008	-0.052	0.139
BB_0807	0.829	0.789	0.258	0.171	0.217	0.266
BB_L11	-0.186	0.036	0.258	0.083	0.253	0.593
BB_0808	0.057	0.055	0.256	0.036	0.127	0.768
BB_0195	0.265	0.072	0.256	-0.016	0.255	0.044
BB_L41	0.274	0.418	0.255	0.163	0.167	0.673
BB_P28-m2	0.747	0.673	0.254	-0.016	0.372	1.043
BB_I19	-0.314	-0.206	0.254	0.009	0.086	0.956
BB_0727	-0.007	0.016	0.253	0.07	0.054	0.127
BB_0826	0.61	0.718	0.252	0.039	0.292	0.631
BB_0041	-0.354	-0.31	0.252	-0.031	0.018	0.516
BB_0298	0.4	0.473	0.251	-0.067	0.11	0.396
BB_0124	1.024	0.908	0.25	0.431	0.625	0.351
BB_0012	0.396	0.521	0.25	0.186	0.249	0.342
BB_E17	0.728	0.73	0.249	0.064	0.139	0.427
BB_F14	0.242	0.716	0.249	0.119	0.3	0.414
BB_G14	0.658	0.682	0.247	0.2	0.277	0.408
BB_0442	0.14	0.377	0.245	0.272	0.671	0.716
BB_0716	0.408	0.458	0.245	0.053	0.145	0.427
BB_0453	0.171	0.159	0.245	0.044	0.046	0.724
BB_A09	0.228	0.13	0.245	0.022	0.116	0.28
BB_0737	0.262	0.19	0.244	0.059	0.068	0.141
BB_0214	0.425	0.256	0.244	-0.051	-0.077	0.117
BB_K22	0.075	0.322	0.243	0.06	0.412	0.684
BB_0373	0.679	0.771	0.242	-0.157	0.369	0.463
BB_0502	-0.697	-0.489	0.242	-0.085	-0.12	-0.138
BB_G08	0.682	0.358	0.241	0.071	0.22	0.855
BB_0722	0.507	0.659	0.24	0.457	0.75	0.932
AF029912 st.IP90 noss	0.427	0.33	0.24	0.286	0.336	0.359
BB_0424	0.63	0.098	0.24	0.002	0.04	0.543
BB_0111	0.009	0.142	0.24	0.042	0.148	0.614
BB_0094	0.034	0.095	0.24	0.117	0.139	0.501
BB_Q73	-0.111	0.354	0.239	0.011	0.143	1.108

BB_0444	0.607	0.604	0.238	0.11	0.337	0.728
BB_0545	0.76	0.641	0.238	0.118	0.312	0.346
BB_0303	0.7	0.574	0.238	0.078	0.239	0.387
BB_N12	-0.115	0.085	0.238	0.137	0.166	0.274
BB_Q39	0.526	0.507	0.236	0.25	0.341	0.324
BB_0346	0.595	0.756	0.235	0.23	0.921	0.574
BB_B13	0.193	0.403	0.235	0.061	0.301	0.678
BB_0045	-0.158	-0.086	0.235	-0.013	0.305	0.275
BB_0675	0.592	0.098	0.234	0.048	0.123	0.37
BB_0397	0.363	0.305	0.233	0.062	0.049	0.275
BB_0042	0.239	0.138	0.233	0.039	0.06	0.288
BB_F17	0.183	0.194	0.233	0.036	-0.007	0.049
BB_Q60#1	-0.034	-0.096	0.233	-0.038	0.035	0.24
BB_A23	0.795	0.964	0.232	0.146	0.344	0.527
BB_J01	0.776	0.64	0.232	-0.009	0.039	0.82
BB_0043	0.174	0.299	0.232	0.144	0.113	0.322
BB_0440	0.75	0.627	0.231	0.187	0.249	0.744
BB_0370	0.032	0.634	0.231	0.003	0.345	0.685
BB_D06	0.318	0.601	0.231	0.171	0.143	0.246
BB_A76	0.159	0.166	0.23	0.048	0.091	0.247
BB_0613 (fcqc)	-0.023	-0.136	0.23	0.004	0.046	0.168
BB_0300	0.762	0.783	0.229	0.089	0.304	0.679
BB_0128	0.079	-0.044	0.229	0.013	0.062	0.06
BB_M39	0.805	0.801	0.228	0.218	0.404	0.67
BB_0504	0.654	0.355	0.228	-0.042	0.171	0.542
BB_0259	-0.05	0.261	0.227	0.165	0.728	0.524
BB_0761	-0.065	0.125	0.227	0.037	0.106	0.452
BB_0136	-0.374	0.122	0.226	-0.046	0.135	0.682
BB_0242	0.411	0.4	0.223	0.091	0.146	0.332
BB_L16	0.356	0.367	0.221	-0.038	0.009	0.496
BB_Q36	0.122	0.224	0.221	0.207	0.264	0.369
BB_0308	0.833	0.995	0.219	0.259	0.298	0.548
BB_0709	0.333	0.331	0.219	0.065	0.023	0.143
BB_0102 . duplicate	0.587	0.604	0.218	0.106	0.201	0.661
BB_A10	0.704	0.813	0.217	0.063	0.18	0.126
BB_0593	0.495	0.446	0.217	0.058	0.211	0.302
BB_H26	0.073	0.37	0.216	0.3	0.53	0.722
BB_0125	-0.199	-0.159	0.216	0.002	0.062	0.227
BB_0672	0.258	0.166	0.215	0.037	0.149	0.46
BB_L23 (fcqc)	-0.2	-0.052	0.215	-0.147	-0.112	0.004
BB_0086	0.471	0.567	0.213	0.152	0.194	0.378
BB_0455	0.338	0.493	0.213	0.033	0.233	0.281
BB_0267	-0.071	0.188	0.212	-0.031	0.307	0.407
BB_0260	-0.241	-0.158	0.209	0.785	1.553	0.162
BB_0499	0.495	0.401	0.209	0.087	0.162	0.256
BB_L30	-0.167	0.069	0.209	0.217	0.549	0.574
BB_L10	0.509	0.578	0.209	0.003	-0.038	0.011
BB_0133	-0.025	-0.095	0.209	-0.025	0.03	0.12
BB_0384	0.642	0.638	0.206	0.161	0.197	0.55
BB_0679	0.494	0.039	0.206	-0.044	-0.031	0.069
BB_Q72	0.484	0.511	0.205	0.838	1	0.672
BB_0060	1.329	1.42	0.205	0.269	0.194	0.114
BB_0537	0.423	0.546	0.205	0.149	0.162	0.428
BB_0118	0.331	0.758	0.204	0.042	0.294	0.315
BB_G03 (fcqc). duplicate	0.395	0.418	0.204	-0.011	-0.001	0.289
BB_0186	0.986	0.971	0.203	0.169	0.113	0.619
BB_0139	0.486	0.949	0.202	0.377	0.261	0.276
BB_0539	0.5	0.684	0.202	0.146	0.203	0.471
BB_G07	0.243	0.376	0.202	0.252	0.407	0.693

BB_0052	0.209	0.332	0.202	0.079	0.241	0.6
BB_0562	0.892	0.936	0.201	0.157	0.151	0.298
BB_0164	0.737	0.595	0.199	0.128	0.187	0.052
BB_R38	0.312	0.631	0.198	0.267	0.463	0.445
BB_0412	0.465	0.411	0.198	0.074	0.284	0.212
BB_0485	0.467	0.447	0.198	-0.006	0.08	0.362
BB_M35	0.08	0.182	0.198	0.305	0.399	0.208
BB_0718	-0.065	0.086	0.198	0.051	0.267	0.569
BB_0647	0.073	0.178	0.198	0.054	0.103	0.127
BB_0311	0.935	0.707	0.197	0.013	0.072	0.06
BB_0387	-0.544	-0.293	0.197	-0.124	-0.105	0.007
BB_D18	0.283	0.185	0.196	0.007	0.157	0.174
BB_0426	0.94	0.829	0.195	0.055	0.168	0.246
BB_0301	0.77	0.627	0.195	0.088	0.067	0.212
BB_0625	0.036	0.495	0.195	0.224	0.44	0.503
BB_0685	0.06	0.247	0.195	0.109	0.294	0.562
BB_J41	0.389	0.489	0.195	-0.05	-0.037	0.062
BB_E16	0.521	0.463	0.194	0.094	0.175	0.173
BB_0578	0.283	0.343	0.194	0.072	0.201	0.093
BB_0190	0.478	0.301	0.193	0.037	0.101	0.428
BB_A12	0.723	0.753	0.192	0.513	0.717	0.429
BB_0454	0.718	0.591	0.192	0.133	0.287	1.007
BB_0276	0.702	0.701	0.192	0.227	0.271	0.537
BB_0063	0.148	0.375	0.191	0.169	0.356	0.197
BB_0636	0.784	0.706	0.19	0.102	0.317	0.372
BB_0016	0.839	0.511	0.19	0.086	0.182	0.17
BB_0818	0.144	0.136	0.19	0.04	0.076	-0.046
BB_0597	0.021	-0.018	0.19	-0.024	0.138	0.189
BB_A15	1.054	0.343	0.189	0.063	0.098	0.619
BB_C01	0.318	0.238	0.189	0.116	0.449	0.371
BB_A33	0.254	0.877	0.188	0.189	0.283	0.543
BB_K03	-0.177	-0.324	0.188	0.249	0.482	0.805
BB_T07-m2	0.416	0.326	0.188	0.009	0.032	0.186
BB_E02	-0.426	-0.204	0.188	-0.038	-0.065	0.142
BB_0770	0.399	0.541	0.187	0.067	0.029	0.217
BB_0292	-0.133	-0.103	0.187	-0.036	0.034	0.389
BB_0050	1.132	1.108	0.186	0.25	0.268	0.453
BB_K17	0.662	0.464	0.186	0.054	0.181	0.724
BB_B08	0.513	0.69	0.186	0.077	0.227	0.553
BB_0665	0.527	0.454	0.186	0.136	0.167	0.507
BB_Q55	0.288	0.451	0.185	0.081	0.366	0.643
BB_0481	0.024	0.363	0.184	0.13	0.188	0.623
BB_0594	0.075	0.308	0.184	0.151	0.311	0.365
BB_0031	0.311	0.385	0.183	0.057	0.115	0.102
BB_0687	0.002	0.2	0.183	0.107	0.092	0.259
BB_O31	0.601	0.595	0.182	0.258	0.468	0.491
BB_J27	0.786	0.921	0.182	0.106	0.115	0.118
BB_I40	0.598	0.715	0.182	0.11	0.134	0.196
BB_I04	0.062	0.327	0.18	0.063	0.171	0.594
BB_0496	0.616	0.374	0.179	0.216	0.412	0.57
BB_0022	0.336	0.507	0.179	0.055	0.099	0.588
BB_T02	0.53	0.652	0.178	-0.014	0.232	0.348
BB_0173	0.613	0.632	0.177	0.217	0.584	0.573
BB_K23	0.277	0.27	0.177	0.189	0.406	0.495
BB_0825 (fcqc)	0.061	-0.049	0.176	-0.022	-0.023	0.317
BB_0035	-0.604	-0.478	0.176	-0.023	0.182	0.543
BB_Q83	0.72	0.711	0.175	0.04	0.143	0.303
BB_0735	0.787	0.683	0.175	0.002	0.109	0.055
BB_0450 (fcqc)	0.23	0.186	0.175	-0.018	-0.074	0.046

BB_0219	1.075	1.034	0.174	0.02	0.049	-0.221
BB_0156	0.307	0.331	0.174	-0.023	0.077	0.081
BB_0141	0.323	0.343	0.174	-0.001	0.051	0.012
BB_0169	0.138	-0.235	0.173	-0.038	-0.019	0.13
BB_B15 (fcqc)	0.202	0.096	0.172	-0.063	-0.11	0.013
BB_0787	-0.381	-0.129	0.172	-0.011	0.031	0.232
BB_G10	-0.373	-0.428	0.172	0.098	0.181	-0.034
BB_A01	1.349	1.247	0.17	0.106	0.266	0.419
BB_F26 (fcqc)	0.305	0.116	0.17	-0.092	-0.072	0.326
BB_N29	0.189	0.068	0.17	0.001	0.084	0.193
BB_I36	0.3	0.379	0.169	0.085	0.188	0.425
BB_Q35-m1	0.727	0.785	0.168	0.147	0.112	0.366
BB_B29 (fcqc)	0.283	0.205	0.168	0.027	0.176	0.189
BB_0621	0.245	0.177	0.168	-0.018	0.052	0.211
BB_0820	0.177	0.171	0.168	0.024	0.109	0.055
BB_0480. duplicate	0.3	0.204	0.167	0.107	0.188	0.701
BB_0586	0.503	0.951	0.166	0.158	0.204	0.33
BB_G34	0.162	0.875	0.165	0.161	0.267	0.689
BB_0471	0.623	0.265	0.165	0.143	0.328	0.49
BB_G12	0.511	0.545	0.162	0.024	0.186	1.452
BB_0382	0.61	0.644	0.162	0.058	0.223	0.556
BB_0707	0.226	0.577	0.161	0.113	0.463	0.73
BB_0726	0.241	0.368	0.161	0.086	0.034	0.101
BB_B24	0.29	0.415	0.16	0.213	0.315	0.312
BB_0081	-0.07	-0.093	0.16	-0.019	-0.027	0.283
BB_0467	0.558	0.538	0.159	0.117	0.182	0.199
BB_0241	0.088	0.144	0.159	0.076	0.078	0.313
BB_0284	-0.055	0.077	0.158	0.417	0.653	0.232
BB_J07	0.133	0.301	0.158	-0.002	0.052	0.511
BB_0429	0.146	0.023	0.158	-0.019	0.08	0.449
BB_0001	0.578	0.53	0.157	0.073	0.237	0.697
BB_0521	0.507	0.451	0.157	0.244	0.297	0.488
BB_0395 (fcqc)	0.079	0.2	0.157	0.033	0.047	0.068
BB_0044	1.17	1.137	0.156	0.654	0.801	1.105
BB_0491	0.596	0.422	0.156	0.125	0.068	0.381
BB_0018	0.242	0.241	0.156	0.083	0.094	0.172
BB_0269	0.433	0.388	0.154	0.103	0.188	0.256
BB_0358	-0.006	-0.041	0.154	-0.004	0.051	0.317
BB_H19	-0.108	-0.121	0.154	-0.042	0.008	0.002
BB_0809	0.218	0.293	0.153	0.023	0.297	0.517
BB_0331	0.345	0.336	0.153	0.084	0.154	0.18
BB_P30	0.051	0.05	0.153	-0.003	0.046	0.655
BB_0457	-0.088	0.076	0.153	0.017	0.231	0.237
BB_0441	0.494	0.36	0.151	0.192	0.42	0.802
BB_0490	0.497	0.351	0.151	0.085	0.088	0.41
BB_0013	0.06	-0.079	0.151	-0.019	0.098	-0.08
BB_E20	0.287	0.459	0.15	0.02	0.343	0.528
BB_0590	0.396	0.271	0.15	0.136	0.139	0.359
BB_R18	0.29	0.165	0.149	0.103	0.189	0.048
BB_0053	0.522	0.532	0.148	0.111	0.271	0.44
BB_0185	0.449	0.631	0.148	0.041	0.251	0.496
BB_0667	0.443	1.106	0.147	0.346	0.403	0.393
BB_A68	0.776	0.796	0.146	0.07	0.229	0.134
BB_E04	0.549	0.157	0.145	0.546	0.836	0.496
BB_0275	0.238	0.423	0.143	0.071	0.187	0.351
BB_0651	0.799	1.037	0.142	0.028	0.265	0.191
BB_0653	0.466	0.596	0.142	0.042	0.019	0.088
BB_0135	0.311	0.219	0.14	0.113	0.218	0.297
BB_0574	0.58	0.762	0.139	0.065	0.191	0.398

BB_0628	0.692	0.754	0.139	-0.012	0.124	0.442
BB_0582	0.572	0.666	0.138	0.063	0.237	0.441
BB_0748	0.268	0.55	0.138	0.022	0.186	0.767
BB_A06	0.243	0.274	0.138	-0.025	0.039	0.025
BB_0374 (fcqc)	-0.188	-0.199	0.138	-0.012	-0.013	0.033
BB_0802	0.589	0.624	0.137	-0.019	0.243	0.516
BB_J03	0.364	0.104	0.136	0.074	0.066	0.464
BB_0393	-0.041	0.258	0.136	0.003	-0.026	0.177
BB_0551	0.21	0.099	0.136	-0.012	-0.069	-0.156
BB_N33	-0.592	-0.618	0.136	-0.087	-0.066	-0.015
BB_0828 . duplicate	-0.168	-0.014	0.135	0.061	0.485	0.639
BB_N30	-0.186	0.02	0.135	-0.017	0.507	0.563
BB_0686	0.662	0.651	0.134	0.066	0.081	0.123
BB_0668	-0.214	-0.431	0.134	-0.033	0.027	0.188
BB_0026	0.499	0.534	0.133	0.145	0.236	0.402
BB_0130	0.443	0.639	0.133	0.125	0.166	0.219
BB_Q38	-0.113	0.204	0.133	0.006	0.417	0.435
BB_0423	0.272	-0.015	0.133	0.05	0.104	0.394
BB_0799	0.966	1.113	0.132	0.19	0.146	0.192
BB_R21	1.02	0.883	0.132	0.108	0.162	0.145
BB_D07	0.117	0.1	0.132	-0.105	-0.066	0.135
BB_0221 (fcqc)	-0.124	-0.27	0.131	-0.07	-0.013	0.068
BB_G29	0.027	0.501	0.13	0.143	0.48	0.639
BB_0758	0.554	0.532	0.13	0.15	0.194	0.219
BB_0092	0.437	0.688	0.129	0.145	0.427	0.592
BB_0813 (fcqc)	0.92	0.886	0.129	0.054	0.226	0.07
BB_D10	0.409	0.575	0.129	0.024	0.351	0.605
BB_P28-m1	0.316	0.255	0.129	0.023	0.434	0.452
BB_0400	0.374	0.471	0.129	0.025	0.094	-0.009
BB_J24	0.557	0.311	0.129	-0.018	0.02	-0.044
BB_F22	-0.091	-0.179	0.129	-0.074	-0.08	-0.3
BB_0699	0.119	0.015	0.128	0.015	0.252	0.072
BB_C11	-0.298	-0.078	0.128	-0.108	0.295	-0.529
BB_0479	0.279	0.293	0.127	0.346	0.46	0.624
BB_B02	0.5	0.694	0.126	0.341	0.478	0.717
BB_U05	0.08	0.237	0.126	-0.009	0.085	0.08
BB_0525	0.431	0.425	0.125	0.932	0.631	0.088
BB_0404	0.424	0.495	0.125	0.167	0.252	0.711
BB_0614 (fcqc)	0.128	0.007	0.125	0.004	-0.021	0.059
BB_0656	0.707	0.572	0.124	0.014	0.166	0.39
BB_J08	-0.178	-0.187	0.123	0.011	0.234	0.68
BB_0305	0.469	0.424	0.122	0.73	0.821	0.43
BB_0772	0.246	0.363	0.122	0.044	0.255	0.308
BB_0246	0.584	0.477	0.122	-0.035	-0.08	-0.155
BB_0196 (fcqc)	-0.158	-0.158	0.122	0.033	0.014	-0.022
BB_A02	-0.122	-0.127	0.122	-0.026	0.006	-0.046
BB_A69-m1	1.003	1.141	0.121	0.163	0.157	0.129
BB_A54	0.477	0.485	0.121	0.081	0.223	0.201
BB_0743	0.069	0.367	0.121	-0.015	0.464	0.467
BB_J29	0.568	0.614	0.12	0.056	0.164	0.004
BB_A26	0.403	0.472	0.12	0.102	0.149	0.018
BB_J06	-0.201	-0.199	0.12	-0.079	-0.049	0.045
BB_0498 (fcqc)	-0.089	0.036	0.119	-0.11	-0.094	0.352
BB_A52-m2	-0.188	-0.024	0.119	-0.091	-0.025	0.174
BB_M29	0.494	0.43	0.116	0.273	0.409	0.54
BB_0788	0.15	0.411	0.116	-0.01	0.442	0.437
BB_0596	0.421	0.527	0.116	0.031	0.267	0.117
BB_E06	0.165	0.11	0.116	-0.023	-0.013	0.054
BB_0768	0.828	0.53	0.115	0.107	0.281	0.202

BB_0549	0.727	0.7	0.115	0.02	0.09	0.267
BB_U04	0.658	0.711	0.114	0.187	0.713	0.674
BB_0306. duplicate	0.335	0.296	0.114	0.121	0.153	0.285
BB_I12	-0.041	-0.163	0.113	-0.062	-0.046	0.055
BB_0552	0.759	0.712	0.112	0.028	0.316	0.124
BB_F05	0.21	0.066	0.112	-0.011	0.063	0.181
BB_0318	0.449	0.723	0.111	0.15	0.255	0.217
BB_0568	0.176	0.377	0.111	0.095	0.253	0.603
BB_L26	0.46	0.341	0.111	-0.006	-0.02	-0.287
BB_0027	0.053	0.304	0.109	0.008	0.011	0.327
BB_0640	-0.037	0.102	0.108	-0.045	-0.053	0.017
BB_0661	0.962	0.392	0.107	0.176	0.231	0.435
BB_0480 . duplicate	0.39	0.577	0.107	0.126	0.251	0.425
BB_Q70	0.051	-0.015	0.107	0.004	0.048	0.135
BB_0174	-0.194	0.041	0.107	-0.055	0.003	0.101
BB_0123	0.009	-0.002	0.107	-0.086	-0.029	-0.013
BB_S36	0.225	0.047	0.106	-0.016	0.119	0.181
BB_N20	0.5	0.599	0.105	0.09	0.306	0.439
BB_D17	0.49	0.504	0.105	0.096	0.014	-0.068
BB_0205	0.027	0.108	0.105	0.097	0.467	0.162
BB_I32	0.561	0.66	0.103	-0.01	-0.075	-0.042
BB_0492	0.434	0.289	0.103	-0.037	-0.058	0.049
BB_A14-m1 (fcqc)	-0.204	-0.195	0.103	-0.027	-0.021	0.095
BB_H36	0.343	0.351	0.101	0.257	0.596	0.295
BB_M37	0.161	0.265	0.101	0.06	0.249	0.35
BB_J39	0.157	0.3	0.101	0.014	0.098	0.059
BB_0252 (fcqc)	-0.492	-0.265	0.101	-0.118	-0.268	-0.481
BB_0025	0.122	0.11	0.1	0.043	0.121	-0.128
BB_0048	0.851	0.838	0.099	-0.026	0.04	0.127
BB_0755	0.258	0.337	0.099	0.039	0.114	0.246
BB_0421	0.212	0.265	0.099	-0.034	0.054	0.099
BB_0064	0.134	0.142	0.099	-0.105	-0.053	0.005
BB_0428	0.927	0.948	0.098	0.06	0.12	0.188
BB_A58	0.334	0.341	0.098	-0.066	-0.085	-0.249
BB_Q20	-0.39	-0.233	0.098	-0.077	-0.064	0.062
BB_0560	0.793	0.652	0.097	0.237	0.449	0.531
BB_0087	0.495	0.722	0.096	0.033	0.191	0.117
BB_0575	-0.241	-0.312	0.096	0.02	0.18	0.257
BB_A17	0.516	0.481	0.095	0.083	0.061	-0.133
BB_C03	0.254	0.236	0.095	0.113	0.177	0.221
BB_J02	-0.2	-0.225	0.095	-0.177	-0.22	-0.274
BB_D12	-0.408	-0.293	0.094	-0.072	-0.081	0.065
BB_0817	0.071	0.11	0.093	0.01	0.172	0.089
BB_B11	-0.051	0.072	0.092	0.123	0.379	0.49
BB_0830. duplicate	0.654	0.516	0.091	0.178	0.301	0.176
BB_0255	0.522	0.43	0.091	0.029	-0.042	0.024
BB_C12	-0.133	0.013	0.091	-0.058	0.034	0.285
BB_0183	-0.084	0.013	0.091	-0.06	-0.003	0.172
BB_Q62	0.678	0.566	0.09	-0.012	0.018	0.138
BB_0102 . duplicate	0.696	0.416	0.09	-0.034	0.035	0.231
BB_0273	0.418	0.488	0.09	-0.05	-0.064	-0.225
BB_I03	0.103	0.032	0.089	-0.024	0.059	-0.107
BB_0740	0.323	0.445	0.088	0.117	0.406	0.758
BB_0838	0.576	0.484	0.088	0.012	0.377	0.302
BB_0605	0.476	0.686	0.088	0.128	0.291	0.137
BB_0733	0.448	0.254	0.088	0.006	0.158	0.103
BB_0329	-0.376	-0.261	0.088	-0.05	0.01	0.085
BB_0662	0.877	0.66	0.087	0.18	0.308	0.675
BB_U09	0.091	0.175	0.087	0.012	0.098	0.193

BB_J30	-0.342	-0.243	0.087	-0.037	-0.011	0.263
BB_0051	0.18	0.273	0.086	0.04	-0.051	-0.023
BB_0723	-0.362	-0.318	0.086	-0.014	0.038	0.234
BB_H14	0.36	0.447	0.085	-0.033	0.045	0.123
BB_J43	-0.08	-0.056	0.085	0.021	0.113	-0.104
BB_L34	-0.47	-0.433	0.085	-0.124	0.014	0.091
BB_F001	0.575	0.407	0.084	0.125	0.148	0.584
BB_G26	0.236	0.182	0.084	0.042	0.131	0.167
BB_0530	0.348	0.339	0.084	0.027	-0.081	-0.136
BB_0780	-0.524	0.067	0.084	-0.115	-0.044	0.131
BB_0751	0.571	0.561	0.083	0.013	-0.021	-0.084
BB_0017	-0.17	0.045	0.083	-0.074	-0.075	0.061
BB_I27	0.076	-0.11	0.083	-0.129	-0.099	-0.083
BB_B18	-0.006	0.098	0.082	0.043	0.291	0.379
BB_E11	-0.067	0.154	0.082	-0.054	0.02	0.174
BB_0262 (fcqc)	-0.178	-0.152	0.082	-0.039	0.001	-0.063
BB_Q43	-0.213	-0.084	0.081	0.082	0.279	0.685
BB_A45	0.354	0.256	0.081	-0.034	0.097	0.051
BB_H10	-0.559	-0.285	0.081	-0.049	-0.01	0.218
BB_0129	0.601	0.495	0.08	0.113	0.108	0.026
BB_J46	0.617	0.66	0.08	-0.089	0.018	-0.008
BB_D11	0.272	0.553	0.08	0.02	0.148	0.081
BB_0556	-0.089	-0.091	0.08	0.015	0.066	0.117
BB_J38 (fcqc)	-0.059	-0.097	0.079	-0.128	-0.084	-0.099
BB_0217	-0.225	-0.103	0.079	-0.143	-0.076	-0.165
BB_0385	0.799	0.823	0.078	0.068	0.121	0.217
BB_O29	0.331	0.175	0.078	0.08	0.026	0.075
BB_0623 (fcqc)	-0.098	-0.163	0.078	-0.014	0.275	0.294
BB_B06	0.199	0.177	0.078	-0.114	-0.087	-0.128
BB_K38	0.139	0.02	0.077	0.014	0.044	-0.012
BB_0216	-0.097	0.005	0.077	0.006	-0.099	-0.092
BB_Q10	-0.764	-0.561	0.077	-0.114	-0.081	0.236
BB_N35	-0.077	0.093	0.076	0.029	0.174	0.616
BB_G30	-0.228	-0.26	0.076	-0.104	-0.024	-0.001
BB_J28	0.294	0.722	0.075	-0.017	0.12	0.093
BB_0766	0.371	0.575	0.075	0.044	0.033	-0.024
BB_0414	-0.03	0.055	0.075	-0.027	0.105	0.171
BB_0368	0.843	0.69	0.074	0.176	0.23	0.323
BB_J32	0.175	0.141	0.074	0.114	0.221	0.459
BB_0398	-0.677	-0.506	0.074	-0.106	-0.138	-0.093
BB_J49	-0.163	-0.169	0.073	0.388	0.65	0.383
BB_J11	0.112	0.11	0.073	-0.074	-0.131	-0.27
BB_0676	-0.011	0.025	0.073	-0.136	-0.1	-0.073
BB_K54	-0.145	-0.246	0.073	-0.03	-0.008	0.036
BB_0261	0.577	0.57	0.072	0.09	0.124	0.168
BB_A61	0.056	0.213	0.072	0.08	0.202	0.413
BB_0003	0.352	0.244	0.072	0.051	0.002	-0.069
BB_0639	0.036	0.113	0.072	-0.078	-0.051	-0.003
BB_0200	0.218	0.201	0.071	0.053	0.096	0.51
BB_P26	0.105	0.103	0.071	-0.119	-0.115	-0.083
BB_D15	0.474	0.522	0.07	-0.152	-0.009	0.117
BB_0225	-0.061	-0.2	0.07	-0.009	-0.006	-0.072
BB_J001 (fcqc)	0.648	0.651	0.069	-0.067	0.031	0.435
BB_0721	0.674	0.647	0.069	0.126	0.084	0.127
BB_0435	0.636	0.586	0.069	0.042	0.234	-0.045
BB_D05	0.134	-0.123	0.069	-0.091	-0.057	0
BB_0236	0.581	0.806	0.068	-0.01	0.018	0.219
BB_0533	-0.531	-0.054	0.068	-0.037	-0.008	0.208
BB_L09	0.8	0.785	0.067	0.049	0.049	-0.006

BB_0822	0.109	0.1	0.067	-0.011	-0.006	-0.044
BB_0816	-0.495	-0.27	0.067	-0.085	-0.107	0.145
BB_0523	0.095	0.242	0.066	-0.031	0.059	0.348
BB_0264	0.375	0.213	0.066	0.016	-0.056	-0.253
BB_0583	0.664	0.553	0.065	0.047	0.109	-0.064
BB_J13	0.142	-0.042	0.065	0.041	-0.011	0.133
BB_0619	0.643	0.772	0.064	0.056	0.121	0.112
BB_0320	0.332	0.642	0.064	0.163	0.295	-0.062
BB_0039	0.371	0.31	0.064	-0.02	0.227	0.03
BB_L12	-0.14	-0.157	0.063	0.075	0.142	0.182
BB_0237	0.722	0.615	0.062	-0.006	0.174	0.088
BB_0528 (fcqc)	-0.106	-0.186	0.062	-0.003	0.053	0.147
BB_0290	-0.027	0.011	0.062	-0.016	0.034	-0.178
BB_D01	-0.222	-0.019	0.062	-0.291	-0.385	0.507
BB_O30	0.518	0.326	0.061	-0.073	0.505	0.446
BB_0354	0.331	0.429	0.061	0.059	0.069	0.124
BB_J25	0.824	0.834	0.06	0.098	0.215	-0.028
BB_A21	-0.01	-0.046	0.06	0.031	0.287	0.049
BB_0089	-0.027	-0.154	0.059	-0.065	-0.093	-0.075
BB_0033	0.209	0.182	0.058	-0.004	0.441	0.058
BB_M33	0.336	0.37	0.057	0.239	0.789	0.131
BB_0085	0.02	0.11	0.057	-0.059	-0.064	0.078
BB_0376	-0.622	-0.379	0.056	-0.099	-0.067	-0.201
BB_R30	-0.061	0.131	0.055	-0.065	-0.039	0.074
BB_T07-m1	0.507	0.471	0.054	0.078	0.098	0.179
BB_E26	0.288	0.244	0.054	-0.02	-0.001	-0.075
BB_G06 (fcqc)	-0.26	-0.237	0.054	-0.036	-0.019	0.124
BB_I43	0.186	0.071	0.053	-0.037	0.004	0.034
BB_F21	-0.027	0.099	0.053	-0.093	-0.106	-0.163
BB_M30	0.222	0.321	0.051	-0.001	0.285	0.307
BB_0634	0.011	-0.255	0.05	0.189	0.551	0.346
BB_I02	0.577	0.602	0.049	0.03	0.068	0.152
BB_0778	0.642	0.518	0.049	0.066	0.031	0.085
BB_0622	0.146	0.145	0.049	0.129	0.226	0.211
BB_B22	0.23	0.364	0.048	-0.001	0.179	0.063
BB_0660	0.075	0.101	0.048	-0.089	-0.003	0.129
BB_J23	0.804	0.657	0.047	0.148	0.3	0.551
BB_J21	0.828	0.803	0.047	0.076	0.164	0.239
BB_0117	0.725	0.691	0.046	0.164	0.224	0.254
BB_0224	0.518	0.515	0.046	0.106	0.18	0.083
BB_O21	1.014	1.005	0.045	0.124	0.224	0.289
BB_0680	0.23	0.259	0.045	-0.076	0.136	0.159
BB_0356	0.136	0.178	0.045	-0.07	-0.007	0.133
BB_0059	0.113	0.114	0.045	-0.098	0.225	-0.089
BB_H08	-0.037	-0.211	0.045	0.004	0.006	0
BB_0509 (fcqc)	-0.254	-0.167	0.045	-0.035	-0.046	0.176
BB_0494	0.394	0.293	0.044	-0.004	0.18	0.242
BB_0671	-0.062	0.209	0.044	-0.032	-0.036	-0.013
BB_0324	0.585	0.572	0.043	0.007	0.154	0.198
BB_0654 (fcqc)	0.114	0.076	0.043	0.057	0.041	0.034
BB_0208	-0.153	0.093	0.042	0.131	0.452	0.653
BB_O42	0.29	0.247	0.042	-0.011	0.069	0.182
BB_I15	-0.067	-0.208	0.042	-0.061	-0.05	-0.179
BB_O20	0.741	0.579	0.041	-0.046	0.147	0.026
BB_I11 (fcqc)	-0.29	-0.202	0.041	-0.12	-0.076	0.063
BB_0413	0.204	0.219	0.04	0.039	0.054	0.178
BB_0810	0.216	0.319	0.039	0.006	0.203	0.121
BB_0630	-0.192	-0.094	0.039	-0.058	-0.066	0.281
BB_U02	-0.224	-0.193	0.039	-0.114	-0.066	-0.102

BB_0244	0.547	0.361	0.038	0.06	0.153	0.079
BB_0577	0.154	0.068	0.037	0	0.001	0.146
BB_0076	-0.021	0.043	0.037	0.028	0.027	-0.056
BB_0120	0.053	-0.077	0.037	0.034	-0.031	-0.007
BB_0103	-0.128	-0.276	0.037	0.038	-0.077	0.008
BB_0291	0.075	-0.084	0.036	0.041	0.174	0.069
BB_0204 (fcqc)	-0.639	-0.524	0.036	-0.076	-0.162	-0.128
BB_0229	0.243	0.183	0.035	-0.043	0.009	0.007
BB_0253 (fcqc)	-0.228	-0.21	0.035	-0.053	-0.044	-0.163
BB_0462	-0.048	-0.151	0.034	0.086	0	-0.344
BB_0159 noss stB31	0.361	0.36	0.033	0.327	0.319	0.144
BB_0313	0.311	0.193	0.032	0.123	0.135	0.357
BB_0218	0.046	0.206	0.032	0.009	0.022	0.066
BB_0819	0.568	0.37	0.031	0.081	0.13	0.151
BB_0192	0.435	0.632	0.031	0.019	0.164	0.014
BB_Q80#1	0.467	0.473	0.031	0.015	0.138	0.076
BB_I31	1.418	1.15	0.03	0.102	0.113	0.141
BB_Q01	0.26	0.327	0.03	0.092	0.124	-0.022
BB_0110	-0.155	0.016	0.03	0.037	0.204	0.099
BB_0345	0.212	0.401	0.029	0.565	0.819	0.371
BB_I29	0.854	1.008	0.029	0.03	0.107	0.328
BB_0501	0.29	0.516	0.029	-0.001	0.048	0.303
BB_0448	0.18	0.11	0.029	-0.066	0.018	0.08
BB_0175	-0.109	-0.144	0.029	-0.01	0.098	0.189
BB_0422	-0.312	-0.171	0.029	-0.006	0.044	0.265
BB_0127	-0.508	-0.251	0.029	-0.059	0.145	0.241
BB_0321	0.625	0.572	0.028	0.032	0.036	-0.043
BB_0202	0.312	0.418	0.028	0.024	0.059	-0.003
BB_L36 (fcqc)	-0.136	0.003	0.028	-0.06	-0.084	-0.11
BB_0779	0.832	0.897	0.027	-0.039	-0.056	-0.002
BB_0250	0.399	0.308	0.026	-0.025	0.036	0.145
BB_0732	0.122	0.318	0.025	0.113	0.315	0.064
BB_0821	0.003	0.113	0.025	-0.045	0.005	0.105
BB_0452 (fcqc)	-0.021	0.141	0.025	-0.06	0.032	-0.115
BB_0767 (fcqc)	-0.324	-0.122	0.025	-0.045	-0.097	-0.126
BB_0319	0.537	0.577	0.022	0.106	0.357	0.592
BB_F04	0.583	0.493	0.022	0.114	0.181	0.029
BB_0165	0.277	0.445	0.022	0.067	0.194	-0.075
BB_0137	0.204	0.108	0.022	-0.011	0.176	0.163
AF517959 noss St IP90	0.219	0.116	0.022	0.022	-0.094	-0.109
BB_0145	-0.539	-0.385	0.022	-0.082	-0.101	-0.126
BB_0702	0.21	0.274	0.021	-0.078	-0.134	-0.159
BB_0332	-0.348	0.007	0.021	-0.097	-0.091	0.006
BB_0149	-0.191	-0.25	0.021	-0.051	0.056	-0.133
BB_0666	0.605	0.537	0.019	0.004	-0.02	0.081
BB_L24	-0.434	-0.54	0.019	-0.088	-0.233	-0.234
BB_D24	-0.271	-0.25	0.017	-0.005	-0.026	-0.18
BB_0753	0.463	0.576	0.016	0.046	0.025	0.09
BB_0830 . duplicate	0.195	0.177	0.016	0.02	0.162	-0.035
BB_0520	-0.072	-0.05	0.016	-0.129	-0.141	-0.109
BB_M40	-0.124	-0.383	0.016	-0.004	-0.014	-0.063
BB_J50-m2	0.327	0.562	0.015	-0.024	0.044	0.002
BB_J15	-0.155	0.011	0.015	-0.006	0.093	0.063
BB_0708	0.649	0.778	0.014	-0.006	0.177	0.408
BB_0681	0.51	0.799	0.014	-0.011	0.275	-0.052
BB_0281	0.444	0.377	0.014	-0.016	0.004	0.231
BB_0585	-0.103	0.011	0.014	0.061	0.2	0.353
BB_0706 (fcqc)	-0.094	-0.188	0.014	-0.106	-0.098	-0.102
BB_0781	-0.404	-0.13	0.014	-0.136	-0.118	-0.106

BB_0228	-0.803	-0.755	0.014	-0.129	-0.013	-0.57
BB_J17	0.45	0.438	0.013	0.205	0.23	0.139
BB_O38	0.613	0.027	0.013	-0.059	0.088	0.395
BB_0558	0.275	0.13	0.013	0.026	0.103	0.059
BB_E10	0.27	0.1	0.013	0.049	0.103	-0.197
BB_0403	0.859	0.468	0.011	0.131	0.062	0.058
BB_0616	-0.153	-0.097	0.011	0.026	0.155	0.203
BB_0309	0.005	-0.091	0.011	-0.038	-0.033	-0.139
BB_G16	0.772	0.721	0.01	0.03	0.038	-0.214
BB_C05	0.23	0.086	0.01	-0.036	0.063	0.487
BB_0470	0.152	0.213	0.01	0.168	0.181	0.105
BB_0571	-0.16	-0.052	0.01	-0.047	0.042	0.003
BB_0608	-0.141	0.01	0.009	0.016	0.203	0.287
BB_F06	-0.225	-0.276	0.009	-0.051	-0.036	-0.139
BB_0206	0.704	0.508	0.008	0.27	0.4	0.576
BB_0170	0.089	0.467	0.007	0.156	0.395	0.246
BB_0379	0.425	0.235	0.007	0.026	0.032	0.167
BB_Q71	0.487	0.562	0.007	-0.086	-0.074	-0.063
BB_0314	-0.032	0.028	0.007	-0.101	-0.021	-0.093
BB_0302	0.247	0.067	0.006	0.051	0.089	-0.035
BB_0461	0.086	0.245	0.006	-0.017	-0.038	-0.003
BB_J22	-0.055	-0.088	0.006	-0.043	-0.123	-0.315
BB_O36	-0.009	0.243	0.005	0.098	0.343	0.728
BB_0272	0.062	0.157	0.005	0.001	-0.017	-0.231
BB_0199	0.11	0.336	0.003	-0.059	0.324	0.251
BB_B07-m2	0.071	0.088	0.002	0.077	0.177	0.183
BB_Q67 (fcqc)	0.234	0.159	0.002	-0.062	0.012	-0.16
BB_0747 (fcqc)	-0.241	-0.072	0.002	-0.162	-0.097	-0.019
BB_A35	-0.072	0.052	0.001	-0.034	0.053	-0.158
BB_E01 (fcqc)	-0.381	-0.167	0.001	-0.084	-0.132	-0.164
OspC Type 8#2 (fcqc)	-0.527	-0.401	0.001	-0.089	-0.161	-0.227
BB_0207	-0.186	-0.07	0	-0.097	-0.176	-0.514
BB_0729	0.187	0.126	-0.001	0.003	0.155	0.011
BB_0598	-0.01	0.162	-0.001	-0.088	0.027	-0.075
BB_I18	0.027	-0.068	-0.001	-0.019	0.003	-0.226
BB_0288	-0.183	-0.148	-0.001	-0.074	0.176	-0.145
BB_0243	-0.55	-0.364	-0.001	-0.009	0.119	0.15
BB_H34	-0.158	-0.065	-0.001	-0.071	-0.111	-0.272
BB_N11	0.167	0.303	-0.002	0.042	0.185	0.128
BB_0473 (fcqc)	0.038	-0.083	-0.002	0.015	0.035	0.239
BB_0678 (fcqc)	-0.168	-0.231	-0.002	0.15	0.121	-0.045
BB_H41	-0.562	-0.388	-0.002	-0.105	-0.115	-0.183
BB_0527	0.56	0.555	-0.003	0.102	0.136	0.312
BB_0143	0.046	-0.024	-0.003	-0.045	-0.003	0.17
BB_0112	-0.022	-0.091	-0.003	-0.082	-0.087	-0.118
BB_0714 (fcqc)	-0.622	-0.463	-0.003	-0.129	-0.057	0.618
BB_0848	0.587	0.593	-0.006	0.032	-0.021	0.111
BB_T01	0.027	0.081	-0.006	0.032	0.169	0.182
BB_0464	-0.176	-0.187	-0.006	-0.081	-0.048	0.114
BB_0357	-0.146	-0.259	-0.006	-0.041	-0.048	-0.026
BB_B23	0.924	0.964	-0.007	-0.052	0.174	-0.045
BB_0741	-0.529	-0.171	-0.007	-0.03	-0.057	0.369
BB_0046	-0.351	-0.206	-0.007	-0.111	-0.067	0.21
BB_F11	0.359	0.578	-0.008	0.216	0.294	0.154
BB_0529	0.236	0.197	-0.008	-0.075	-0.06	-0.136
BB_M14	-0.531	-0.563	-0.008	0.18	0.157	0.209
BB_0604	-0.414	-0.219	-0.008	-0.079	0.066	-0.067
BB_0401	0.354	0.522	-0.01	0.116	0.298	0.406
BB_0193	-0.041	0.027	-0.01	-0.105	-0.082	0.003

BB_0287	0.117	-0.137	-0.011	-0.008	-0.007	-0.033
BB_A62-m2	-0.293	-0.042	-0.011	-0.163	-0.164	-0.171
BB_0776	0.608	0.374	-0.012	0.056	0.147	0.034
BB_0342	-0.048	0.012	-0.012	0.013	0.095	0.017
BB_K18	-0.149	-0.052	-0.013	-0.093	-0.083	0.087
BB_0002	-0.448	-0.146	-0.013	-0.074	-0.085	-0.114
BB_S38	-0.212	-0.009	-0.014	-0.001	0.303	0.751
BB_0180	-0.039	0.045	-0.014	-0.057	-0.177	-0.134
BB_0333	0.577	0.522	-0.015	0.381	0.372	1.685
BB_0179	-0.146	-0.128	-0.015	-0.06	0.045	-0.023
BB_K36 (fcqc)	0.018	-0.127	-0.015	-0.09	-0.116	-0.346
BB_0617	0.185	0.105	-0.016	-0.007	0.013	-0.137
BB_0643	0.206	0.08	-0.016	-0.08	-0.086	-0.105
BB_F12	-0.229	-0.15	-0.016	-0.131	-0.084	-0.044
BB_0432	0.208	0.053	-0.019	0.103	0.325	0.27
BB_0061	0.092	-0.086	-0.02	-0.063	-0.027	-0.104
BB_0587	0.022	-0.033	-0.02	-0.063	0.098	-0.248
BB_O18	0.743	0.624	-0.021	0.089	0.13	0.148
BB_Q02	0.029	0.033	-0.021	-0.036	-0.044	-0.151
BB_L35 (fcqc)	0.084	0.118	-0.021	-0.078	-0.149	-0.202
BB_0030	-0.183	-0.018	-0.022	0.038	0.065	0.048
BB_H40 (fcqc)	-0.371	-0.212	-0.022	-0.039	-0.001	-0.187
BB_0731	-0.632	-0.465	-0.023	-0.09	-0.014	0.42
BB_0692	0.334	0.367	-0.024	0.07	0.039	0.143
BB_0637	0.46	0.426	-0.026	0.059	0.158	-0.082
BB_0317	0.215	0.237	-0.026	-0.038	0.149	-0.146
BB_D09	0.908	0.754	-0.028	0.149	0.296	0.489
BB_0420	0.37	0.286	-0.028	0.131	0.411	0.125
BB_0138	0.621	0.592	-0.028	0.034	0.034	-0.112
BB_A65	0.39	0.27	-0.028	-0.027	-0.012	-0.088
BB_0827	-0.319	-0.191	-0.028	0.06	0.28	0.161
OspC Type 8 (fcqc)	0.516	0.569	-0.029	0.066	0.234	0.094
BB_0644	0.404	0.398	-0.029	-0.076	0.008	0.043
BB_0524	0.003	0.108	-0.029	-0.028	0.003	-0.024
BB_0181	-0.059	-0.13	-0.029	-0.134	-0.058	0.048
BB_0580	-0.236	-0.159	-0.029	-0.063	-0.046	-0.023
BB_H09 (fcqc)	-0.124	0.086	-0.03	0.047	0.413	0.354
BB_0007 (fcqc)	-0.094	-0.089	-0.03	0	-0.002	-0.095
BB_0238	-0.17	-0.183	-0.031	-0.101	-0.105	-0.141
BB_H27	0.473	0.647	-0.032	0.085	0.256	0.581
BB_0646 (fcqc)	0.126	0.13	-0.032	-0.033	-0.071	-0.048
BB_0336	-0.673	-0.505	-0.032	-0.086	-0.058	-0.077
BB_0132	0.54	0.31	-0.033	-0.009	0.248	-0.013
BB_0295	-0.449	-0.496	-0.033	0.013	0.018	0.215
BB_0828. duplicate	-0.072	0.143	-0.034	-0.018	0.492	0.43
BB_K29	-0.23	-0.242	-0.035	-0.093	-0.131	0.062
BB_Q86	-0.623	-0.273	-0.035	-0.121	-0.073	0.013
BB_H09.1	0.64	0.778	-0.036	0.062	0.112	0.291
BB_0116	0.78	0.714	-0.036	0.042	0.104	-0.125
BB_B05	0.113	-0.008	-0.036	0.07	0.219	-0.194
BB_A51	0.496	0.501	-0.037	-0.026	0.045	-0.087
BB_A47	0.469	0.457	-0.037	0.02	-0.044	-0.032
BB_0201	-0.002	0.185	-0.037	0.048	0.432	0.009
BB_B16	0.081	-0.044	-0.037	-0.181	-0.048	-0.082
BB_H28 (fcqc)	0.103	0.229	-0.038	-0.011	-0.008	-0.035
BB_0690	0.307	0.138	-0.038	0.021	0.034	-0.286
BB_0327	-0.562	-0.541	-0.038	-0.07	-0.02	0.347
BB_J37	-0.479	-0.351	-0.038	-0.028	-0.042	-0.129
BB_J14	0.483	0.266	-0.039	0.656	0.77	0.141

BB_L13	0.232	0.05	-0.039	-0.021	-0.022	-0.104
BB_G32	-0.061	0.025	-0.04	0.073	0.147	0.416
BB_0526	0.43	0.351	-0.04	-0.068	0.079	-0.198
BB_K45	-0.165	-0.002	-0.04	-0.065	-0.09	-0.175
BB_Q77	-0.442	-0.388	-0.04	0.039	0.081	-0.163
BB_0040 (fcqc)	-0.301	-0.286	-0.04	-0.033	-0.109	-0.268
BB_0610	0.335	0.233	-0.041	0.041	0.16	0.245
BB_R43	0.267	0.125	-0.041	-0.02	-0.034	-0.234
BB_I33 (fcqc)	-0.423	-0.432	-0.041	-0.11	-0.14	-0.122
BB_J20	-0.742	-0.516	-0.041	-0.142	-0.223	-0.073
BB_A37	0.494	0.412	-0.042	0.15	0.293	0.458
BB_0592	0.797	0.631	-0.042	0.134	0.024	-0.192
BB_0075	-0.006	-0.069	-0.042	0.003	0.071	0.015
BB_0759	-0.199	-0.015	-0.042	-0.049	-0.01	-0.068
BB_0843 (fcqc)	0.279	0.178	-0.043	-0.041	-0.084	0.168
BB_0762	-0.604	-0.443	-0.043	-0.085	-0.086	0.098
BB_L03	-0.125	-0.063	-0.044	1.71	1.343	-0.385
BB_0542	0.762	0.783	-0.044	0.122	0.222	0.408
BB_N40	0.081	-0.147	-0.044	-0.051	0.005	-0.089
BB_D14 (fcqc)	-0.173	-0.057	-0.044	-0.068	0.025	-0.022
BB_J12	-0.153	-0.08	-0.044	-0.034	-0.122	-0.093
BB_0131	-0.178	0.035	-0.045	0.017	0.167	0.128
BB_0155	0.103	0.189	-0.047	0.038	0.228	0.154
BB_0293	0.181	-0.285	-0.047	-0.034	-0.124	-0.134
BB_0486	-0.365	-0.455	-0.047	-0.116	-0.059	0.002
BB_Q05	0.3	0.327	-0.048	0.123	0.13	0.304
BB_0736	-0.453	-0.013	-0.048	-0.141	-0.118	-0.086
BB_0652	0.309	0.12	-0.049	0.019	0.12	0.215
BB_0852 (fcqc)	-0.194	-0.259	-0.049	-0.061	-0.075	-0.111
BB_0396	-0.364	-0.346	-0.049	-0.076	-0.135	0.018
BB_J16	0.316	0.295	-0.05	-0.073	0.05	-0.436
BB_0014	-0.244	-0.323	-0.051	-0.013	0.238	0.095
BB_0011	-0.241	-0.127	-0.051	-0.054	-0.027	0.19
blank	-0.989	-0.674	-0.051	-0.169	-0.235	-0.26
BB_E03	0.572	0.575	-0.052	-0.036	-0.097	-0.218
BB_0198	-0.29	-0.252	-0.052	-0.107	-0.132	-0.377
BB_0515	-0.298	-0.207	-0.052	-0.11	-0.19	-0.515
BB_0032	0.22	0.175	-0.053	-0.049	0.126	-0.053
BB_E14	-0.28	-0.208	-0.053	-0.154	-0.033	-0.298
BB_G02	0.654	0.099	-0.054	-0.042	0.062	0.154
BB_0419	0.226	0.382	-0.054	0.018	-0.086	0.054
BB_0645	0.115	0.185	-0.054	-0.057	-0.006	-0.207
BB_0705	-0.204	-0.128	-0.054	-0.05	-0.031	0.196
BB_M24	-0.664	-0.477	-0.054	-0.184	-0.25	-0.296
BB_0738	0.206	0.076	-0.055	0.035	0.187	0.082
BB_0489	-0.454	-0.383	-0.055	-0.115	-0.134	-0.114
BB_0315	0.713	0.593	-0.057	-0.002	-0.01	-0.102
BB_H38	-0.815	-0.64	-0.057	-0.196	-0.181	-0.011
BB_0121	-0.115	-0.089	-0.058	-0.077	-0.039	-0.244
BB_P35	0.062	0.388	-0.059	0.238	0.539	0.418
BB_A14-m2	0.544	0.778	-0.059	0.077	0.097	0.077
BB_0569	-0.048	-0.123	-0.059	0.015	0.224	-0.02
BB_0601	0.305	0.25	-0.06	0.006	0.042	0.207
BB_0566	0.629	0.845	-0.06	-0.153	-0.199	-0.727
BB_J31	-0.361	-0.292	-0.06	-0.057	0.015	-0.171
BB_A05-m1 (fcqc)	-0.354	-0.25	-0.06	-0.129	-0.123	-0.046
BB_F08	-0.065	-0.123	-0.062	-0.022	-0.091	-0.136
BB_Q76	-0.314	-0.408	-0.062	0.013	0.088	0.101
BB_I08	0.376	0.323	-0.063	-0.006	-0.11	-0.154

BB_0256	-0.613	-0.458	-0.063	-0.122	-0.148	-0.018
BB_0106	0.642	0.644	-0.065	0.113	0.239	-0.085
BB_I16 (fcqc)	0.263	0.083	-0.065	0.057	0.099	-0.057
BB_0038	0.28	0.261	-0.066	0.006	-0.029	0.079
BB_C08	0.092	0.166	-0.066	-0.096	0.005	-0.231
BB_H04	-0.098	-0.107	-0.067	-0.134	-0.079	-0.246
BB_0561	-0.214	-0.284	-0.067	-0.12	0.039	-0.139
BB_J18 (fcqc)	-0.545	-0.377	-0.067	-0.045	-0.144	-0.164
BB_H25	0.544	0.508	-0.068	0.009	-0.011	-0.251
BB_M32	-0.153	-0.127	-0.068	-0.163	-0.195	-0.495
BB_J10	0.584	0.495	-0.069	0.134	0.123	-0.116
BB_0516	-0.428	-0.208	-0.07	-0.039	-0.033	0.4
BB_0615	-0.893	-0.612	-0.07	-0.111	-0.11	0
BB_0754	-0.113	-0.037	-0.071	-0.11	-0.142	-0.164
BB_O25	-0.033	-0.267	-0.071	-0.123	-0.162	-0.318
BB_0082	-0.264	-0.248	-0.072	0.012	0.068	0.022
BB_0570	-0.322	-0.377	-0.072	-0.071	-0.032	-0.014
BB_0833 (fcqc)	-0.367	-0.383	-0.072	-0.053	-0.148	-0.496
BB_A39	-0.78	-0.607	-0.072	-0.121	-0.137	-0.095
BB_D25 (fcqc)	-0.807	-0.554	-0.072	-0.142	-0.164	-0.249
BB_L37	-0.155	-0.13	-0.073	-0.038	0.056	0.411
BB_0095 (fcqc)	-0.248	-0.303	-0.073	-0.074	-0.088	-0.184
BB_0595	0.581	0.471	-0.074	0.034	0.077	0.15
BB_0600	0.499	0.377	-0.074	0.099	0.261	-0.04
BB_F28	-0.307	-0.191	-0.074	-0.03	-0.131	-0.186
BB_A67	-0.326	-0.321	-0.074	-0.148	0.021	-0.345
BB_L21	0.483	0.399	-0.075	0.008	0.071	-0.101
BB_0339	0.614	0.416	-0.077	0.013	0.062	-0.131
BB_0618	0.287	0.33	-0.077	-0.03	0.102	-0.056
BB_0493	0.102	0.259	-0.077	-0.093	0.01	0.06
BB_0853	-0.01	-0.231	-0.077	-0.138	-0.084	-0.118
BB_0466	-0.577	-0.419	-0.077	-0.113	-0.096	0.11
BB_0674	-0.033	-0.055	-0.078	-0.145	-0.131	-0.234
BB_K08	-0.074	-0.091	-0.078	-0.102	-0.124	-0.425
BB_0304 (fcqc)	-0.471	-0.409	-0.078	-0.158	-0.228	-0.314
BB_0632	-0.815	-0.718	-0.079	-0.04	0.097	0.069
BB_A36 noss st B31 (fcqc)	-0.616	-0.49	-0.079	-0.121	-0.183	-0.336
BB_0683	0.764	0.726	-0.08	-0.058	0.034	-0.228
BB_0257	0.323	0.271	-0.08	-0.047	0.137	-0.117
BB_0447	-0.041	-0.009	-0.08	-0.049	0.052	-0.181
BB_N41	-0.267	-0.199	-0.08	-0.108	-0.046	-0.174
BB_0446	0.042	-0.113	-0.081	0.039	0.264	0.175
BB_U08	-0.098	-0.214	-0.082	-0.048	-0.022	-0.15
BB_0029	0.322	0.424	-0.083	0.105	0.04	0.116
BB_0090	0.222	0.348	-0.083	-0.04	-0.075	0.008
BB_0824	-0.067	0.123	-0.083	-0.055	0.004	0.033
BB_0782	-0.055	0.025	-0.084	0.051	0.108	0.249
BB_0097	0.411	0.445	-0.085	-0.012	0.073	0.005
BB_M03	-0.115	-0.147	-0.085	-0.126	-0.124	-0.167
BB_H22	0.117	-0.015	-0.086	-0.074	0.025	-0.169
BB_0849	-0.082	-0.138	-0.086	-0.12	0.025	-0.144
BB_0803 (fcqc)	-0.293	-0.593	-0.087	-0.074	-0.117	-0.272
BB_A49	-0.153	-0.162	-0.088	-0.081	-0.051	-0.104
BB_J04	0.234	0.225	-0.089	-0.02	-0.023	-0.078
BB_Q46	0.045	0.153	-0.089	-0.044	0.029	0.15
BB_0611	0.124	0.017	-0.089	-0.023	0.051	0.046
BB_0248	-0.138	-0.227	-0.089	-0.033	0.177	0.031
BB_Q45	-0.053	0.05	-0.09	-0.147	-0.048	0.172
BB_K51	-0.036	0.167	-0.09	-0.071	0.013	-0.281

BB_0297	0.011	-0.116	-0.09	-0.151	-0.084	0.053
BB_0274	0.097	0.157	-0.091	0.187	0.547	0.068
BB_0362	0.423	0.454	-0.091	0.024	-0.018	-0.14
BB_0775	0.009	-0.062	-0.091	0.054	-0.086	-0.048
BB_K33	-0.514	-0.318	-0.091	-0.117	-0.15	0.016
BB_0806 (fcqc)	-0.429	-0.266	-0.091	-0.148	-0.128	-0.162
BB_K35	-0.148	-0.017	-0.092	-0.124	-0.152	0.002
BB_0113	-0.134	0.03	-0.092	-0.113	-0.15	-0.238
BB_N22	-0.457	-0.318	-0.093	0.171	0.527	0.082
BB_S35	0.142	0.052	-0.093	-0.131	-0.071	-0.322
BB_I07	-0.079	-0.165	-0.093	-0.055	-0.099	-0.078
BB_A28	-0.407	-0.334	-0.093	-0.109	-0.151	-0.193
BB_0576	0.441	0.531	-0.094	-0.064	0.224	-0.09
BB_0474	0.246	0.201	-0.095	0.012	0.094	-0.187
BB_P33	-0.298	-0.209	-0.096	-0.104	-0.068	-0.211
BB_0019	-0.174	-0.295	-0.096	-0.14	-0.095	-0.357
BB_A32	-0.03	-0.046	-0.097	-0.098	-0.088	-0.17
BB_U06	0.126	-0.027	-0.098	0.038	0.178	0.066
BB_0697	0.292	0.239	-0.099	-0.032	0.141	0.068
BB_O13	0.258	-0.031	-0.099	-0.076	-0.044	-0.171
BB_Q80#2	-0.236	-0.401	-0.099	-0.136	-0.016	-0.327
BB_0340	0.154	0.027	-0.101	-0.073	0.048	-0.196
BB_0550	0.088	-0.011	-0.101	-0.111	-0.087	-0.178
BB_Q11	-0.159	-0.27	-0.102	-0.158	-0.202	0.424
BB_0153	-0.296	-0.15	-0.102	-0.072	0.049	-0.232
BB_0469	0.656	1.323	-0.103	-0.1	-0.107	-0.335
BB_0801	-0.691	-0.445	-0.103	0.237	0.37	1.23
BB_0134	0.441	0.313	-0.104	0.138	0.239	0.124
BB_0840	0.287	0.253	-0.104	0.016	0.085	0.045
BB_I21	-0.374	-0.278	-0.104	-0.054	-0.144	-0.301
BB_0677	-0.23	-0.113	-0.105	-0.062	0.088	-0.015
BB_B25	0.703	0.738	-0.106	-0.093	-0.024	-0.232
BB_0482 (fcqc)	0.052	-0.139	-0.106	0.046	-0.159	-0.224
BB_0546	-0.352	-0.293	-0.106	-0.13	-0.115	0.11
BB_0531	-0.344	-0.222	-0.106	-0.121	-0.194	-0.087
BB_0557	-0.42	-0.242	-0.108	-0.119	-0.13	0.038
BB_I25 (fcqc)	-0.831	-0.593	-0.108	-0.117	-0.139	-0.199
BB_0263	0.26	0.18	-0.109	-0.065	0.008	-0.166
BB_U10	-0.325	-0.423	-0.109	-0.113	-0.173	-0.171
BB_0425	0.064	-0.006	-0.11	-0.136	-0.13	-0.156
BB_0760	-0.052	-0.112	-0.11	-0.06	-0.158	-0.224
BB_Q68	0.55	0.47	-0.111	0.11	0.139	-0.069
BB_K11	0.518	0.377	-0.111	0.02	0.059	-0.079
BB_0316	0.316	0.37	-0.111	-0.039	-0.038	-0.153
BB_0717	0.264	0.156	-0.112	-0.085	-0.082	-0.288
BB_L29 (fcqc)	0.328	0.194	-0.114	-0.026	0.028	-0.061
BB_K10	0.054	0.188	-0.115	-0.123	-0.081	0.122
BB_0157	0.321	0.262	-0.115	-0.084	-0.073	-0.273
BB_E13	-0.201	-0.396	-0.115	-0.059	-0.099	-0.25
BB_I24	0.611	0.513	-0.116	0.035	-0.096	-0.177
BB_0609	0.001	0.036	-0.116	-0.108	-0.14	-0.267
BB_I05	-0.521	-0.415	-0.118	-0.093	-0.098	-0.214
BB_0197	-0.048	0.037	-0.119	-0.086	-0.076	-0.054
BB_0847	0.161	0.293	-0.12	-0.032	-0.038	-0.003
BB_0693	0.04	-0.021	-0.12	0.021	0.131	0.094
BB_0488	-0.155	-0.149	-0.12	-0.042	-0.081	-0.14
BB_0366	-0.339	-0.381	-0.12	-0.048	-0.075	-0.199
BB_J44	-0.018	-0.018	-0.122	-0.018	-0.004	0.093
BB_0700	0.016	-0.061	-0.122	0.031	0.112	-0.094

BB_E04.1	0.126	0.208	-0.122	-0.103	-0.065	-0.241
BB_I14	-0.405	-0.187	-0.122	-0.004	0.189	-0.042
BB_E32	-0.159	-0.076	-0.122	-0.166	-0.132	-0.047
BB_K25.1	-0.115	-0.063	-0.122	-0.053	-0.177	-0.302
BB_0579 (fcqc)	0.229	0.197	-0.123	-0.064	0.057	0.063
BB_0349	0.24	0.088	-0.123	0.026	0.132	-0.007
BB_0367 (fcqc)	-0.198	-0.154	-0.123	-0.117	-0.08	-0.278
BB_Q59	0.06	-0.246	-0.124	-0.032	-0.021	-0.182
BB_Q56 (fcqc)	-0.393	-0.362	-0.124	-0.097	-0.116	-0.261
BB_K06	-0.531	-0.33	-0.125	-0.103	-0.119	-0.243
BB_E08	0.375	0.313	-0.126	-0.026	0.076	-0.098
BB_I01	0.693	0.708	-0.127	0.014	0.026	-0.385
BB_H11 (fcqc)	-0.155	-0.242	-0.127	-0.09	-0.147	-0.435
BB_K28	-0.448	-0.325	-0.127	-0.207	-0.227	-0.363
BB_0351	0.189	0.022	-0.13	0.028	0.232	-0.029
BB_0837 (fcqc)	-0.846	-0.622	-0.13	-0.173	-0.205	-0.272
BB_0624	0.212	0.045	-0.131	-0.008	-0.004	0.016
BB_B03	0.144	0.355	-0.132	-0.107	0.11	-0.107
BB_F31	-0.719	-0.503	-0.133	-0.111	-0.106	0.128
BB_0369	0.195	-0.042	-0.134	0.034	0.4	-0.012
BB_S43	-0.276	-0.244	-0.134	0.053	0.229	0.053
BB_0495	-0.125	-0.121	-0.134	-0.147	-0.216	-0.183
BB_0325	0.216	0.022	-0.135	0.023	0.162	-0.047
BB_0375	-0.393	-0.201	-0.136	0.064	-0.03	-0.095
BB_I09	-0.133	-0.233	-0.136	-0.085	-0.194	-0.349
BB_0189	-0.669	-0.571	-0.136	-0.134	-0.105	-0.124
BB_S32	-0.799	-0.708	-0.136	-0.15	-0.237	-0.31
BB_0177	0.443	0.367	-0.137	0.083	0.097	0.231
BB_0696	-0.257	-0.099	-0.137	0.363	0.441	0.063
BB_P36	-0.294	-0.063	-0.137	-0.089	0.007	0.331
BB_0049	-0.222	-0.181	-0.137	-0.078	-0.015	-0.003
BB_0172	0.32	0.455	-0.138	-0.009	0.077	-0.081
BB_0701	0.005	-0.227	-0.138	-0.005	-0.038	0.081
BB_O05	0.025	-0.089	-0.138	-0.148	-0.098	-0.481
BB_Q78	-0.417	-0.314	-0.138	-0.124	-0.166	-0.028
BB_H33 (fcqc)	-0.604	-0.604	-0.139	-0.099	-0.201	-0.301
BB_0449	0.417	0.388	-0.141	0.223	0.246	0.195
BB_K04	-0.293	-0.409	-0.141	-0.136	-0.191	-0.232
BB_0299	-0.473	-0.421	-0.141	-0.118	-0.031	-0.236
BB_0249	0.37	0.443	-0.143	-0.11	-0.051	-0.139
BB_0115	0.022	0.191	-0.143	0.003	-0.03	0.112
BB_0720	0.01	-0.13	-0.143	-0.032	0.085	-0.044
BB_D08	-0.204	0.076	-0.145	-0.111	-0.047	-0.178
BB_0765	-0.273	-0.3	-0.146	-0.007	-0.041	-0.082
BB_0211	-1.039	-0.899	-0.146	0.135	0.382	0.062
BB_0750	0.117	0.135	-0.147	-0.098	-0.116	-0.221
BB_Q47 (fcqc)	-0.068	-0.134	-0.147	-0.078	-0.176	-0.309
BB_0160	-0.125	-0.109	-0.148	-0.098	-0.003	-0.212
BB_0505	0.018	-0.086	-0.148	-0.101	-0.14	-0.352
BB_P32	-0.408	-0.26	-0.148	-0.04	0.078	-0.048
BB_0487	-0.256	-0.192	-0.15	-0.072	-0.021	-0.139
BB_0483 (fcqc)	-0.577	-0.399	-0.15	-0.082	-0.105	-0.03
BB_L04	-0.46	-0.555	-0.15	-0.207	-0.224	-1.089
BB_0056	-0.664	-0.632	-0.15	-0.296	-0.419	-0.835
BB_0166	0.042	-0.208	-0.152	0.019	0.084	-0.065
BB_A63 (fcqc)	-0.222	-0.392	-0.152	-0.089	-0.199	-0.308
BB_0459	0.557	0.602	-0.153	0.135	0.185	-0.034
BB_L20	0.703	0.608	-0.154	-0.041	-0.033	-0.114
BB_0514	-0.14	-0.123	-0.154	-0.003	0.332	0.002

BB_0734	0.011	-0.211	-0.154	-0.058	0.08	-0.025
BB_S28	-0.071	-0.366	-0.154	0.004	0.077	0.033
BB_0591 (fcqc)	-0.489	-0.571	-0.154	-0.076	-0.153	-0.237
BB_0416	-0.018	0.05	-0.156	0.023	0.156	0.083
BB_F02	-0.275	-0.282	-0.158	-0.131	-0.154	0.176
BB_0417	-0.13	-0.055	-0.158	-0.075	-0.17	-0.312
BB_E31 . duplicate	-0.797	-0.576	-0.158	-0.174	-0.154	-0.104
BB_B04	0.251	0.261	-0.159	0	0.012	-0.129
BB_0407	0.047	0.032	-0.159	0.002	-0.079	-0.261
BB_K09	-0.263	-0.233	-0.16	-0.079	-0.089	-0.306
BB_0191	-0.341	-0.443	-0.161	-0.084	-0.099	-0.048
BB_H29	-0.827	-0.537	-0.161	-0.238	-0.291	-0.5
BB_0057	0.513	0.575	-0.162	0	0.1	-0.092
BB_0408	0.027	0.04	-0.162	0.002	-0.021	-0.151
BB_K16	-0.179	-0.248	-0.162	-0.111	-0.103	-0.361
BB_A60	-0.381	-0.307	-0.162	-0.057	0.029	-0.313
BB_B01	-0.014	0.048	-0.163	-0.042	-0.005	-0.05
BB_C07	-0.31	-0.271	-0.164	-0.132	-0.18	-0.35
BB_0258 (fcqc)	-0.594	-0.478	-0.164	-0.166	-0.231	-0.389
BB_0503	-0.539	-0.61	-0.165	-0.07	-0.041	0.044
BB_Q58	-0.228	-0.365	-0.166	-0.061	0.03	-0.195
BB_0072	-0.56	-0.376	-0.166	-0.086	0.156	-0.102
BB_E24	-0.506	-0.395	-0.166	-0.122	-0.143	-0.335
BB_H23	-0.521	-0.252	-0.166	-0.155	-0.164	-0.456
BB_K15	-0.639	-0.364	-0.167	-0.043	0.067	0.029
BB_0477	-0.349	-0.212	-0.167	-0.121	-0.212	-0.198
BB_0607	0.15	0.055	-0.168	0.067	0.254	0.002
BB_A31	-0.153	-0.213	-0.168	-0.016	-0.013	-0.084
BB_0641	0.045	-0.014	-0.168	-0.087	-0.152	-0.562
BB_G11	-0.303	-0.316	-0.171	0.151	0.361	-0.192
BB_O33	-0.429	-0.225	-0.171	-0.249	-0.217	-0.039
BB_S26	0.003	-0.06	-0.172	-0.11	-0.16	-0.421
BB_0633	-0.58	-0.585	-0.172	-0.078	-0.018	-0.042
BB_Q09	-0.151	-0.077	-0.173	-0.063	0.009	-0.139
BB_0784	-0.29	-0.359	-0.173	-0.104	-0.091	-0.14
BB_E27	-0.47	-0.431	-0.173	-0.154	-0.141	-0.386
BB_0746 (fcqc)	-0.233	-0.27	-0.175	-0.142	-0.272	-0.536
BB_0150 (fcqc)	-0.153	0.005	-0.177	0.017	0.03	0.069
BB_H35	-0.208	-0.193	-0.177	-0.098	-0.218	-0.433
BB_T03	-0.321	-0.271	-0.179	-0.15	-0.092	-0.107
BB_D21. duplicate	-0.364	-0.074	-0.179	-0.146	-0.048	-0.344
BB_I26	-0.485	-0.179	-0.181	-0.101	0.011	-0.115
BB_0188	-0.559	-0.411	-0.181	-0.063	-0.052	-0.233
BB_0232 (fcqc)	-0.49	-0.259	-0.181	-0.129	-0.195	-0.484
BB_J35	-0.671	-0.534	-0.181	-0.074	-0.134	-0.148
OspC_Type3 (fcqc)	-0.273	-0.448	-0.182	-0.102	-0.127	-0.273
BB_0213	-0.195	-0.005	-0.183	-0.075	-0.007	-0.048
BB_0099	-0.207	-0.267	-0.183	-0.118	-0.04	-0.186
BB_O41	0.294	0.013	-0.184	-0.109	-0.094	0.094
BB_0006	-0.178	-0.038	-0.185	-0.054	0.129	0.052
BB_0695	-0.513	-0.323	-0.186	-0.139	-0.185	-0.09
BB_0774	-0.431	-0.336	-0.186	-0.2	-0.249	-0.347
BB_Q64	-0.364	-0.296	-0.187	0.501	0.494	0.137
BB_A43	0.161	0.162	-0.187	0.027	-0.009	-0.138
BB_R33	-0.396	-0.208	-0.187	0.012	0.077	-0.096
BB_0355	-0.387	-0.328	-0.187	-0.224	-0.337	-0.608
BB_L19	0.389	0.239	-0.188	-0.058	-0.086	-0.32
BB_0388	-0.187	-0.105	-0.188	-0.028	0.162	-0.119
BB_H16	0.02	-0.074	-0.189	-0.058	-0.223	-0.377

BB_N05	-0.312	-0.194	-0.189	-0.097	-0.133	-0.351
BB_0541	-0.501	-0.082	-0.19	-0.045	-0.051	0.391
BB_0322 (fcqc)	-0.484	-0.39	-0.19	-0.168	-0.171	-0.355
BB_I28	0.314	0.31	-0.191	-0.235	-0.246	-1.062
BB_Q08	0.453	0.434	-0.192	0.055	0.024	-0.204
BB_0437	-0.615	-0.455	-0.192	-0.131	-0.026	0.066
BB_0058	-0.597	-0.283	-0.194	0.006	0.095	-0.107
BB_0497	-0.25	-0.2	-0.194	-0.112	-0.139	-0.319
BB_F07 (fcqc)	-0.569	-0.359	-0.195	-0.16	-0.226	-0.398
BB_K21	0.538	0.541	-0.196	0.062	0.134	0.225
BB_0353 (fcqc)	-0.271	-0.25	-0.197	-0.131	-0.204	-0.357
BB_E19	-0.318	-0.156	-0.198	-0.168	-0.193	-0.305
BB_0100	-0.329	-0.383	-0.2	-0.091	-0.115	-0.188
BB_0711	-0.04	-0.265	-0.202	-0.035	-0.067	-0.083
BB_0240	-0.379	-0.238	-0.202	-0.116	-0.179	-0.279
BB_0476	-0.272	-0.074	-0.203	-0.106	-0.069	-0.162
BB_J33	-0.132	-0.115	-0.203	-0.094	-0.012	-0.353
BB_0703	-0.761	-0.501	-0.205	-0.04	-0.098	-0.073
BB_R39	-0.643	-0.635	-0.205	-0.182	-0.148	-0.133
BB_0212	-0.387	-0.496	-0.207	-0.13	-0.091	-0.328
BB_0456	0.583	0.455	-0.208	0.041	-0.064	-0.244
BB_0363	-0.032	-0.013	-0.208	-0.059	0.009	-0.248
BB_0176	-0.807	-0.654	-0.21	-0.178	-0.191	-0.197
BB_0638	0.59	0.485	-0.212	0.099	0.114	-0.112
BB_R40	0.04	0.17	-0.212	0.195	0.4	-0.194
BB_0572	-0.133	-0.229	-0.212	-0.033	-0.062	-0.137
BB_D04	-0.344	-0.173	-0.213	-0.144	-0.212	-0.401
BB_A44	-0.338	-0.233	-0.215	-0.095	-0.065	-0.013
BB_0405 (fcqc)	-0.125	0	-0.216	-0.06	-0.041	-0.167
BB_L22	-0.402	-0.454	-0.216	0.046	0.255	-0.043
BB_I23	-0.686	-0.445	-0.216	-0.105	-0.144	-0.028
BB_0080	-0.011	-0.088	-0.217	-0.116	-0.058	-0.438
BB_0849.2	-0.454	-0.505	-0.217	-0.221	-0.202	-0.293
BB_0433	-0.135	-0.006	-0.218	-0.116	-0.112	-0.124
BB_0763	-0.079	-0.069	-0.22	-0.09	0.129	-0.291
BB_0833	0.058	-0.119	-0.221	-0.019	0.121	-0.278
BB_0285	-0.287	-0.178	-0.221	-0.093	-0.014	0.03
BB_Q57	-0.734	-0.739	-0.221	-0.289	-0.407	-0.903
BB_0785	-0.079	-0.206	-0.224	-0.204	-0.28	-0.758
BB_0430	0.146	0.048	-0.226	-0.082	0.069	-0.049
BB_0712	-0.279	-0.307	-0.226	-0.069	-0.006	-0.115
BB_I30	0.503	0.416	-0.227	0.116	0.223	0.058
BB_0323	0.148	0.016	-0.227	-0.118	-0.21	-0.591
BB_E18	-0.405	-0.524	-0.227	-0.239	-0.068	-0.636
BB_N32	-0.177	-0.199	-0.228	-0.116	-0.036	-0.391
BB_H17	-0.402	-0.327	-0.228	-0.112	-0.058	-0.209
BB_0793	-0.188	-0.201	-0.23	-0.032	0.135	0.051
BB_J45	-0.544	-0.59	-0.23	-0.188	-0.224	-0.437
BB_Q19	0.294	0.336	-0.231	0.086	0.006	-0.026
BB_B20	-0.485	-0.428	-0.231	-0.036	-0.036	-0.33
BB_0797 (fcqc)	-0.371	-0.385	-0.232	-0.035	0.166	0.518
BB_I17	-0.352	-0.262	-0.236	-0.162	-0.255	-0.404
BB_0812 (fcqc)	-0.282	-0.31	-0.238	0.059	-0.133	-0.614
BB_0364	-0.365	-0.225	-0.241	-0.077	-0.013	-0.24
BB_0091	-0.352	-0.379	-0.242	-0.19	-0.127	-0.488
BB_K14	-0.03	0.104	-0.243	-0.071	-0.087	-0.011
BB_N37#1	-0.5	-0.573	-0.244	-0.189	-0.289	-0.582
BB_F16	-0.143	-0.197	-0.245	-0.113	-0.132	-0.352
BB_0009	-0.399	-0.294	-0.246	-0.052	0.014	-0.136

BB_F29 (fcqc)	-0.417	-0.449	-0.246	-0.208	-0.236	-0.379
BB_0104 (fcqc)	-0.449	-0.516	-0.247	-0.1	-0.154	-0.237
BB_0626	0.351	0.111	-0.248	0.03	0.112	-0.05
BB_0020	0.085	0.144	-0.249	0.064	0.224	0.147
BB_D02 (fcqc)	-0.037	-0.125	-0.25	-0.025	0.062	-0.012
BB_0391	-0.344	-0.013	-0.25	-0.193	-0.222	-0.24
BB_0326	-0.73	-0.71	-0.251	-0.151	0.075	0.2
BB_P29	-0.016	-0.024	-0.252	0.136	0.251	-0.004
BB_0411	-0.181	-0.127	-0.253	-0.196	-0.217	-0.393
BB_0846	-0.155	-0.117	-0.258	0.18	0.459	-0.281
BB_A42	-0.098	0.25	-0.258	-0.092	-0.055	-0.23
BB_Q61	-0.117	-0.14	-0.258	-0.079	-0.173	-0.358
BB_H15	-0.051	-0.005	-0.261	-0.157	-0.197	-0.422
BB_0689 (fcqc)	-0.276	-0.237	-0.263	-0.139	-0.258	-0.569
BB_A29	-0.655	-0.45	-0.265	-0.132	-0.154	-0.099
BB_0659	-0.417	-0.402	-0.27	-0.126	-0.026	-0.41
BB_G01	-0.346	-0.245	-0.27	-0.294	-0.443	-0.912
BB_K30	0.005	-0.195	-0.271	-0.066	-0.209	-0.612
BB_G04	0.311	0.403	-0.272	0.015	-0.022	-0.293
BB_E30	-0.293	-0.181	-0.272	-0.1	-0.034	-0.263
BB_0101	-0.402	-0.222	-0.272	-0.097	-0.234	-0.409
BB_0500	-0.318	-0.368	-0.273	-0.191	-0.224	-0.363
BB_B10	0.161	0.038	-0.276	0.028	0.163	-0.084
BB_S39	-0.125	0.015	-0.277	-0.069	0.02	0.116
BB_0410	0.235	0.169	-0.281	0.017	0.112	-0.404
BB_0728	-0.117	-0.006	-0.282	-0.095	0.062	0.011
BB_A55. duplicate	-0.355	-0.259	-0.282	-0.163	-0.151	-0.239
BB_A22 (fcqc)	-0.47	-0.458	-0.282	-0.16	-0.179	-0.27
BB_0122 (fcqc)	-0.448	-0.663	-0.282	-0.157	-0.162	-0.231
BB_0510	-0.671	-0.585	-0.282	-0.166	-0.177	-0.184
BB_0518	-0.764	-0.669	-0.282	-0.172	-0.204	-0.609
BB_K31	-0.023	0.006	-0.283	-0.094	-0.111	-0.288
BB_0392	-0.209	-0.123	-0.288	-0.251	-0.043	-0.476
BB_0650	-0.315	-0.25	-0.288	-0.232	-0.204	-0.374
BB_0168	-0.225	0.216	-0.289	-0.026	0.069	0.084
BB_Q66	-0.111	-0.165	-0.29	-0.153	-0.188	-0.326
BB_0335	-0.629	-0.463	-0.291	-0.175	-0.3	-0.576
BB_K24.1	-0.482	-0.453	-0.295	-0.105	-0.128	-0.141
BB_0465	-0.125	-0.16	-0.3	-0.193	-0.217	-0.625
BB_0222	-0.282	-0.225	-0.3	-0.096	-0.271	-0.596
BB_K40	0.305	0.359	-0.301	0.267	0.628	0.116
BB_E15	-0.587	-0.348	-0.304	-0.114	-0.126	-0.24
BB_O32	-0.241	-0.326	-0.304	-0.186	-0.24	-0.643
BB_0804	0.152	-0.093	-0.305	-0.068	-0.145	-0.566
BB_K02	-0.364	-0.335	-0.307	-0.033	0.133	-0.181
BB_0463	-0.396	-0.27	-0.307	-0.178	-0.194	-0.488
BB_C04	-0.254	-0.243	-0.308	-0.201	-0.273	-0.536
BB_0372	0.128	-0.002	-0.31	-0.034	0.054	-0.092
BB_0823	-0.194	-0.344	-0.31	-0.119	-0.112	-0.25
BB_0069	0.058	-0.08	-0.313	0.004	0.091	-0.529
BB_D001	-0.055	-0.204	-0.313	-0.135	-0.233	-0.524
BB_A53	0	0.149	-0.314	0.29	0.434	0.03
BB_0088	-0.301	-0.225	-0.315	-0.022	0.086	-0.253
BB_0725	-0.222	-0.271	-0.317	-0.264	-0.352	-0.857
BB_A71	-0.155	-0.094	-0.323	-0.23	-0.348	-0.93
BB_L17	0.466	0.318	-0.324	-0.044	0.005	-0.352
BB_S42	-0.031	-0.142	-0.324	-0.077	-0.105	-0.424
BB_0460 (fcqc)	-0.341	-0.287	-0.324	-0.136	-0.32	-0.528
BB_A30	-0.26	-0.239	-0.325	-0.288	-0.252	-0.831

BB_G09	0.014	-0.22	-0.326	-0.056	-0.054	-0.421
BB_E22	-0.456	-0.316	-0.337	-0.177	-0.197	-0.419
BB_0658	-0.517	-0.252	-0.339	-0.259	-0.346	-0.617
BB_F27	-1.115	-0.867	-0.339	-0.303	-0.326	-0.379
BB_0835	-0.473	-0.448	-0.34	-0.168	-0.224	-0.467
BB_0829	0.088	-0.271	-0.344	-0.016	0.002	-0.305
BB_0182	-0.273	-0.288	-0.345	-0.015	0.005	-0.22
BB_0436	-0.944	-0.77	-0.348	-0.192	-0.113	-0.167
BB_0719	0.039	0.105	-0.358	-0.09	0.065	-0.203
BB_I41	-0.389	-0.511	-0.361	-0.123	-0.164	-0.135
BB_U03	-0.355	-0.237	-0.361	-0.12	-0.328	-0.555
BB_0394	0.089	-0.024	-0.362	-0.1	-0.223	-0.599
BB_0109	-0.098	-0.285	-0.362	-0.122	-0.191	-0.585
BB_F20	-0.392	-0.376	-0.364	-0.228	-0.342	-0.414
BB_0777	-0.017	0.069	-0.366	-0.22	-0.397	-0.777
BB_A08	0.126	0.063	-0.369	-0.116	-0.047	-0.132
BB_Q49	-0.407	-0.47	-0.369	-0.014	0.263	-0.19
BB_0067	-0.471	-0.275	-0.371	-0.207	-0.205	-0.423
BB_0544	-0.261	-0.127	-0.376	-0.145	-0.174	-0.479
BB_B26	-0.454	-0.489	-0.376	-0.164	-0.082	-0.636
BB_0227	-0.103	-0.06	-0.378	-0.187	-0.159	-0.285
BB_0657	-0.319	-0.547	-0.381	-0.259	-0.292	-0.692
BB_0055	-0.23	-0.409	-0.383	-0.256	-0.34	-0.912
BB_D22	-0.482	-0.527	-0.386	-0.14	-0.287	-0.871
BB_0350 (fcqc)	-0.341	-0.375	-0.39	-0.156	-0.188	-0.283
BB_K27	-0.307	-0.186	-0.391	-0.083	-0.094	-0.244
BB_G21	-0.94	-0.869	-0.392	-0.133	-0.179	-0.312
BB_0842	-0.632	-0.608	-0.393	-0.274	-0.369	-0.783
BB_F30	-0.191	-0.328	-0.395	-0.104	-0.141	-0.478
BB_0434	-0.611	-0.346	-0.397	-0.203	-0.222	-0.38
BB_J51	-0.416	-0.447	-0.402	-0.149	-0.095	-0.376
BB_0231	-0.153	-0.208	-0.402	-0.258	-0.336	-0.869
BB_0739	-0.955	-0.704	-0.402	-0.248	-0.19	-0.438
BB_F14.1	-0.286	-0.468	-0.406	-0.08	-0.147	-0.466
BB_0068	-0.361	-0.35	-0.407	-0.199	-0.241	-0.709
BB_0254	-1.209	-0.942	-0.41	-0.197	-0.047	-0.154
BB_D13	-0.476	-0.402	-0.415	-0.243	-0.407	-0.736
BB_0669	-0.326	-0.319	-0.416	-0.218	-0.226	-0.827
BB_L08	-0.352	-0.563	-0.418	-0.259	-0.301	-0.432
BB_E05	0.138	0.204	-0.419	-0.138	-0.172	-0.392
BB_F23	-0.271	-0.33	-0.419	-0.214	-0.257	-0.75
BB_H24	-0.377	-0.426	-0.419	-0.239	-0.269	-0.633
BB_0756	-0.298	-0.302	-0.421	-0.094	-0.036	-0.424
BB_0226	-0.451	-0.254	-0.421	-0.106	-0.124	-0.263
BB_0283	-0.158	-0.344	-0.422	-0.167	-0.21	-0.616
BB_L32	-0.307	-0.359	-0.424	-0.218	-0.212	-0.651
BB_F24	-0.505	-0.454	-0.424	-0.17	-0.144	-0.767
BB_0631 (fcqc)	-0.527	-0.332	-0.425	-0.148	-0.179	-0.411
BB_0386	-0.402	-0.558	-0.427	-0.19	-0.25	-0.281
BB_O14	-0.451	-0.542	-0.428	-0.13	-0.143	-0.223
BB_0371	-0.522	-0.517	-0.436	-0.274	-0.305	-0.932
BB_0771	-0.547	-0.547	-0.439	-0.183	-0.191	-0.14
BB_0402	-0.359	-0.532	-0.442	-0.142	-0.156	-0.412
BB_0538	-0.346	-0.225	-0.447	-0.372	-0.457	-1.389
BB_A40	-0.052	-0.072	-0.448	-0.046	-0.053	-0.294
BB_0171	0.262	0.188	-0.45	-0.142	-0.252	-0.734
BB_L07	-0.123	-0.05	-0.453	-0.264	-0.374	-0.785
BB_0105	-0.148	-0.225	-0.455	-0.17	-0.298	-0.886
BB_0789	0.775	0.407	-0.46	0.014	0.131	-0.066

BB_0786	-0.143	-0.27	-0.471	-0.223	-0.283	-0.772
BB_0508	-0.085	-0.28	-0.473	0.026	0.095	-0.164
BB_0715	-0.821	-0.698	-0.474	-0.276	-0.27	-0.658
BB_M36	-0.178	-0.137	-0.479	-0.129	-0.129	-0.378
BB_0381	-0.273	-0.25	-0.48	-0.131	-0.023	-0.324
BB_0077	-0.537	-0.65	-0.481	-0.22	-0.294	-0.203
BB_S44	-0.287	-0.366	-0.485	0.04	-0.113	-1.042
BB_0187	-0.011	-0.121	-0.486	-0.022	-0.154	-0.484
BB_L43	-0.26	-0.099	-0.489	-0.094	-0.011	-0.66
BB_0511	-0.301	-0.144	-0.493	-0.197	-0.184	-0.348
BB_J51	0.011	0.077	-0.499	-0.057	-0.128	-0.526
BB_G20	-0.625	-0.42	-0.509	-0.266	-0.321	-0.994
BB_0247	-0.442	-0.392	-0.514	-0.307	-0.488	-0.954
BB_0036	-0.712	-0.698	-0.515	-0.264	-0.211	-0.889
BB_0167	-0.846	-0.107	-0.517	-0.324	-0.427	-1.043
BB_0438	-0.498	-0.679	-0.517	-0.3	-0.363	-1.482
BB_H12	-0.24	-0.173	-0.522	-0.173	-0.235	-0.591
BB_0047	-0.345	-0.427	-0.524	-0.294	-0.506	-1.11
BB_0146	-0.604	-0.341	-0.525	-0.29	-0.264	-0.702
BB_R07	-0.257	-0.364	-0.528	-0.271	-0.412	-0.914
BB_G22	-0.927	-0.771	-0.534	-0.253	-0.277	-0.683
BB_0294	-0.448	-0.584	-0.538	-0.33	-0.536	-1.214
BB_0841	-0.454	-0.414	-0.546	-0.373	-0.364	-1.355
BB_0270	0.321	0.228	-0.55	0.061	-0.218	-0.84
BB_L42	-0.708	-0.483	-0.555	-0.301	-0.281	-1.203
BB_Q60#2	-0.723	-0.475	-0.555	-0.462	-0.513	-1.402
BB_A56	0.181	0.115	-0.558	-0.132	-0.056	-0.477
BB_L01	-0.181	-0.141	-0.558	-0.069	-0.183	-0.761
BB_K26	-0.467	-0.596	-0.563	-0.193	-0.397	-1.034
BB_L06	-0.574	-0.555	-0.567	-0.403	-0.443	-1.48
BB_0606	-0.936	-0.545	-0.577	-0.415	-0.562	-1.208
BB_O09	-0.23	-0.147	-0.579	-0.321	-0.342	-1.081
BB_U07	-0.346	-0.66	-0.583	-0.181	-0.146	-0.552
BB_0773	-0.217	-0.001	-0.6	-0.333	-0.558	-1.114
BB_0245	-0.439	-0.465	-0.604	-0.262	-0.458	-1.191
BB_0266	-0.439	-0.343	-0.606	-0.246	-0.355	-0.991
BB_M06	-0.263	-0.293	-0.609	-0.298	-0.455	-1.443
BB_J26	-0.503	-0.633	-0.616	-0.36	-0.491	-1.548
BB_M07	0.32	0.217	-0.618	-0.265	-0.301	-1.003
BB_0642	-0.944	-0.588	-0.625	-0.238	-0.196	-0.651
BB_0015	-0.362	-0.436	-0.643	-0.254	-0.366	-1.159
BB_0151	-0.263	-0.433	-0.662	-0.223	-0.34	-0.89
BB_A38	-0.902	-0.718	-0.668	-0.247	-0.09	-0.981
BB_D19	-0.467	-0.674	-0.682	-0.245	-0.449	-1.108
BB_J42	-0.881	-0.692	-0.691	-0.38	-0.39	-0.829
BB_0612	-0.495	-0.473	-0.698	-0.415	-0.407	-1.376
BB_0184	-0.606	-0.468	-0.698	-0.344	-0.353	-1.445
BB_0154	-0.429	-0.388	-0.721	-0.269	-0.176	-0.84
BB_M08	-0.664	-0.669	-0.722	-0.35	-0.437	-1.438
BB_M04	-0.801	-0.677	-0.736	-0.441	-0.456	-1.441
BB_0805	-0.489	-0.585	-0.75	-0.31	-0.403	-1.316
BB_R41	-0.902	-0.989	-0.761	-0.394	-0.403	-1.063
BB_R08	-0.335	-0.496	-0.763	-0.184	-0.308	-1.19
BB_0152	-0.671	-0.636	-0.774	-0.303	-0.618	-1.403
BB_0093	-0.7	-0.49	-0.782	-0.315	-0.365	-1.173
BB_0343	-0.411	-0.383	-0.793	-0.349	-0.374	-1.469
BB_A41	-0.534	-0.688	-0.809	-0.357	-0.357	-1.535
BB_0084	-0.495	-0.64	-0.865	-0.286	-0.255	-0.453
BB_O06	-0.704	-0.698	-0.896	-0.345	-0.369	-1.664

BB_G23	-0.386	-0.421	-0.906	-0.354	-0.373	-1.388
BB_K37	-0.71	-0.792	-0.914	-0.406	-0.533	-1.589
BB_0691	-0.856	-0.793	-0.927	-0.296	-0.167	-1.404
BB_N42	-0.664	-0.693	-0.929	-0.426	-0.45	-1.692
BB_G17	-1.053	-0.792	-0.947	-0.55	-0.682	-1.881
BB_R44	-1.103	-0.884	-0.956	-0.522	-0.592	-1.665
BB_0540	-0.591	-0.569	-0.975	-0.305	-0.442	-1.671
BB_0021	-0.501	-0.796	-1	-0.343	-0.48	-1.524
BB_Q82	-0.451	-0.633	-1.01	-0.394	-0.691	-1.967
BB_0730	-0.797	-0.839	-1.057	-0.469	-0.541	-1.941
BB_0178	-1.155	-0.83	-1.076	-0.489	-0.514	-2
BB_G19	-2	-2	-2	-2	-2	-2
